# Mapping neural activity during naturalistic visual and memory search

**DOI:** 10.3389/fnhum.2026.1812919

**Published:** 2026-07-09

**Authors:** Joaquin E. Gonzalez, Markus Bauer, Anthony J. Ries, Juan E. Kamienkowski, Matias J. Ison

**Affiliations:** 1 Laboratorio de Inteligencia Artificial Aplicada, Instituto de Ciencias de la Computación (Universidad de Buenos Aires – Consejo Nacional de Investigaciones Científicas y Técnicas), (C1428EGA), Buenos Aires, Argentina; 2 Departamento de Física (Facultad de Ciencias Exactas y Naturales, Universidad de Buenos Aires), Buenos Aires, Argentina; 3 School of Psychology, University of Nottingham, Nottingham, United Kingdom; 4 U.S. Army DEVCOM Army Research Laboratory, Aberdeen, MD, United States; 5 Departamento de Computación (Facultad de Ciencias Exactas y Naturales, Universidad de Buenos Aires), (C1428EGA), Buenos Aires, Argentina; 6 Maestría de Explotación de Datos y Descubrimiento del Conocimiento (Universidad de Buenos Aires), (C1428EGA), Buenos Aires, Argentina

**Keywords:** eye movements, free-viewing, hybrid search, magnetoencephalography, memory load, neuronal oscillations, target detection, deconvolution

## Abstract

In everyday life, individuals often search for one of several items stored in memory. This cognitive process, known as hybrid search, is critical for tasks like navigating using landmarks. While the behavioral aspects of hybrid search have been extensively studied, the underlying neural mechanisms remain less understood. In this study, we combined concurrent magnetoencephalography (MEG) and eye movement recordings to investigate the oscillatory and evoked neural dynamics supporting hybrid search under naturalistic viewing conditions. Twenty-one participants (12 males, 9 females) performed a free-viewing task involving visual search for targets embedded in memory (hybrid search) across structured, context-rich scenes. Time-Frequency analyses revealed specific neural signatures during memory encoding, retention, and visual search. During encoding and retention, posterior alpha-band power decreased with memory load, and frontoparietal beta-band activity scaled with memory load during visual search. By aligning MEG signals to eye movement events and applying source reconstruction, we identified an early visually evoked lambda response, localized to V1, followed by a distributed P3m component, with maximum activation in the right inferior parietal lobe, that discriminated target from distractor fixations. These findings were further confirmed by a deconvolution approach to account for overlapping neural signals linked to eye movements. These results show that distinct oscillatory and evoked signatures track the dynamic brain responses during hybrid search. Alpha-band modulation reflects heightened perceptual and mnemonic demands, whereas beta-band increases index cognitive control engaged during search. The fixation-locked responses highlight how classic electrophysiological signatures, such as target-related components, generalize to free viewing conditions. Together, these findings demonstrate how oscillatory and evoked responses dynamically support hybrid search under naturalistic viewing conditions, revealing how memory, attention, and visual processing interact during active vision.

## Introduction

A fundamental premise in cognitive neuroscience is that using simple stimuli -designed to minimize confounds-, offers the building blocks for understanding complex real-world behavior. This approach has yielded insights across domains including attention and decision-making (Posner and Petersen, 1990; Buschman and Miller, 2007; Gold and Shadlen, 2007). However, there is little empirical evidence on how these processes -including mechanisms of attentional selection, memory encoding and retrieval, and visual processing- operate during complex behavior in natural viewing behavior.

In visual search, experimental paradigms often rely on artificial stimuli on uniform backgrounds. Neural studies of attention commonly restrict eye movements -using brief presentations (Desimone and Duncan, 1995) or requiring covert search (Çukur et al., 2013) to avoid M-EEG signal artifacts (Carl et al., 2012; Muthukumaraswamy, 2013). However, this overlooks the ubiquity of eye movements in natural vision. While controlled visual search studies (Treisman and Gelade, 1980) have provided key insights, visual search under real-world conditions remains largely unexplored (Peelen and Kastner, 2014). Recent studies combining eye-tracking with neural recordings have started to identify brain signatures of cognitive processes across less constrained viewing conditions. Within the EEG domain, a growing number of studies have explored fixation-related neural responses across a range of cognitive tasks, including visual search involving characters (Kamienkowski et al., 2012; Körner et al., 2014; Brouwer et al., 2017; Hiebel et al., 2018), detecting faces in crowds (Kaunitz et al., 2014), identifying objects in natural scenes (Devillez et al., 2015), and examining covert and overt attention (Kulke et al., 2016). More recently, this approach has been extended to dynamic contexts such as virtual navigation (Stankov et al., 2021), and movie viewing (Nentwich, 2022). Despite MEG’s superior spatial resolution compared to EEG (Muthukumaraswamy, 2013), few studies have employed concurrent MEG and eye tracking, leaving a gap in our understanding of spatiotemporal dynamics during naturalistic visual behavior. Here, we focus on hybrid search, a common and ecologically relevant form of search in which individuals look for any of several memorized targets within visual environments (Schneider and Shiffrin, 1977; Wolfe, 2012; Barbosa et al., 2024). Hybrid search involves memory encoding, when potential targets are learned, and active visual search, where perceptual input is matched against stored representations.

Neuronal oscillations have been widely associated with attention, memory, and perceptual decision-making (Fries, 2005; Rohenkohl and Nobre, 2011; Bauer et al., 2014). In working memory, oscillatory activity recorded via M-EEG and intracranial methods shows frequency-specific links to memory representations (Axmacher et al., 2010; Roux and Uhlhaas, 2014). While early studies focused on theta and gamma rhythms (Jensen and Lisman, 1998), increasing evidence implicates alpha and beta oscillations in memory formation and maintenance (Hanslmayr and Staudigl, 2014; Staudigl et al., 2017). Alpha-band activity typically varies with memory load. Increases in posterior alpha have been linked to the suppression of irrelevant information (Jensen and Mazaheri, 2010; Klimesch, 2012), while decreases have been observed when perceptual detail must be actively maintained (van Ede et al., 2017; van Ede, 2018; Proskovec et al., 2019). These oscillatory dynamics during encoding are thought to shape the quality of memory traces, raising the question of how such neural signatures relate to later memory performance.

A common approach to addressing this question involves assessing amplitude or synchrony during encoding and examining how they relate to whether that memory trace is later successfully remembered or not (Staudigl and Hanslmayr, 2013). The underlying hypothesis is that successfully encoded memory traces are reinstated during retrieval. While oscillatory markers of successful recognition have been widely reported in the literature, most studies have employed paradigms with fixed gaze, limiting their ecological validity (Hanslmayr and Staudigl, 2014). Interestingly, a recent free-viewing memory study reported that phase-locking of alpha oscillations just prior to saccades predicted successful memory encoding (Staudigl et al., 2017), suggesting that oscillatory mechanisms may be closely tied to active visual exploration during memory formation.

Invasive electrophysiology studies in primates actively exploring natural scenes have revealed transient power increases in beta oscillations from LFP signals in the primary visual cortex shortly after fixation onset (Maldonado et al., 2008; Ito et al., 2011). In tasks involving eye movements, beta oscillations have been linked to communication between dorsolateral prefrontal cortex and superior colliculus, a key structure in the oculomotor system (Chan et al., 2015). During a free-viewing visual search task, Pesaran and colleagues observed robust beta-band coherence, supporting the involvement of a frontoparietal circuit in decision-making processes (Pesaran et al., 2008). Converging evidence from (Stoll et al., 2016) further linked beta oscillations to cognitive control mechanisms. Given that hybrid search requires the active maintenance of multiple target representations, increasing memory load is expected to place greater demands on cognitive control, potentially reflected in enhanced beta-band activity.

The neural mechanisms underlying target detection in visual search have been extensively studied using electrophysiological and neuroimaging approaches. One well-established neural signature of target detection is the P3 component (and its magnetic counterpart, P3m), which emerges around 300–600 ms after stimulus onset and is strongly linked to the cognitive effort underlying target identification (Polich, 2007). This response has been widely observed across various paradigms, including oddball and discrimination tasks (Herrmann and Knight, 2001; Kok, 2001; Linden, 2005; Nieuwenhuis et al., 2011) and free-viewing visual search tasks (Devillez et al., 2015; Kamienkowski et al., 2012; Kaunitz et al., 2014; Ries et al., 2016; Hiebel et al., 2018). Simultaneous EEG and fMRI recordings have reported widespread brain activation related to the P3, with potential bilateral generators in the temporal–parietal junction, posterior parietal cortex, inferior parietal and temporal lobes, premotor and motor area, and the anterior intraparietal sulcus (Bledowski et al., 2004; Ragazzoni et al., 2019). Similarly, intracranial recordings from neurosurgical patients have identified P3 generators in widespread regions, including the hippocampus, superior temporal sulcus, ventrolateral prefrontal cortex, and intraparietal sulcus (Halgren et al., 1998).

Together, these findings suggest that oscillatory dynamics and evoked responses reflect core cognitive mechanisms underlying hybrid search. As most evidence comes from simplified paradigms, the building blocks identified in such settings remain disconnected from how they operate during complex behavior. Here, we combined MEG with eye-tracking to investigate fixation-related activity and task-related oscillations during a free-viewing hybrid search task. We tested four hypotheses: (H1) Given that context-rich scenes require high perceptual detail, posterior alpha oscillations will attenuate with increasing memory load; (H2) Alpha/beta power and phase during encoding will predict memory performance; (H3) Because hybrid search requires maintaining multiple target representations, we predict that higher memory load will amplify beta-band activity as a marker of cognitive control; (H4) Fixations on targets will elicit a P3m component, generalizing classic electrophysiological signatures to visual tasks under naturalistic viewing conditions. By investigating how oscillatory and evoked responses support hybrid search under ecologically valid conditions, we aim to identify specific neural mechanisms that operate during complex behavior, offering insights toward ecologically valid models of cognition.

## Materials and methods

### Data acquisition

#### Participants

Twenty-one participants (12 males, 9 females) between the ages of 19 and 45 years (mean: 28.6, SD: 6.4) participated in this study. Participants were given an inconvenience allowance for their participation and provided written consent form after being instructed on every aspect of the experiment. The study was approved by the University of Nottingham School of Psychology Ethics Panel (ethics approval: F1349).

#### Experimental task

**Figure 1:**
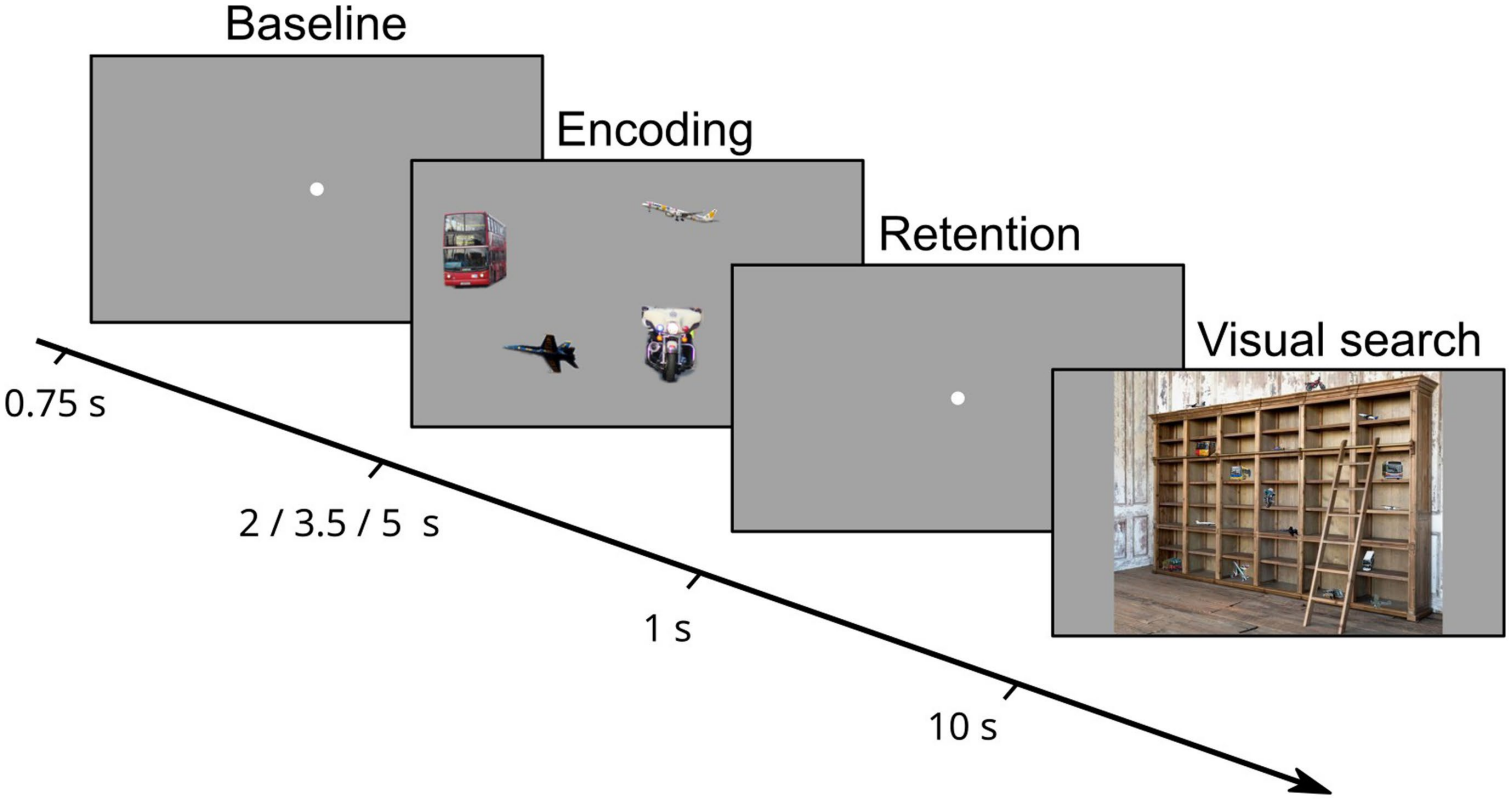
Overview of the experimental task. Each trial consisted of four sequential screens: An initial fixation cross, a memory encoding screen (items enlarged here for clarity), a retention fixation cross, and a context-rich scene for free-viewing visual search. Images were adapted from the COCO dataset (Lin et al., 2014; https://cocodataset.org/, CC BY 4.0) and ImageNet (Deng et al., 2009; http://www.image-net.org/, non‑commercial research license).

The experiment consisted of an all-new mapping (i.e., target changes every trial) hybrid search paradigm of 7 blocks of 30 trials each with an average duration of approximately 54 min. The stimuli were presented on a projected screen of approximately 38 cm width, at an average distance of 61 cm, which corresponds approximately to 34° of visual angle. Each trial was composed of a fixation dot of 750 ms, followed by a memorization/encoding screen. During this screen, the participants were presented with 1, 2 or 4 items corresponding to the memory set size (MSS), and the duration was 2, 3.5 or 5 s, respectively. During this time, participants were asked to memorize the items by freely exploring the screen. This was followed by a retention period of 1 s presenting a fixation dot, and finally, a search screen consisting of an image on which 16 items were embedded (Figure 1). On half of the trials (“target present”), one of these items had previously been presented on the encoding screen; on the other half (“target absent”), none of the items in the search screen had been present during memorization. The search screen had a maximum duration of 10 s; during this time, participants could freely explore the screen and respond if any of the presented items during the encoding period were present or absent by pressing a button with the right/left thumb on a button pad. Participants could respond at any time during the search, which would end the trial. After each trial, there was a 1-s pause to rest. Participants had the choice to take a break between blocks.

In each search image (1,280 × 1,024 pixels), 16 individual items (objects, animals or full-body humans) were superimposed on a real-world background scene (depicting outdoor scenes, e.g. forest, and indoor scenes, e.g. shelf; see Figure 1 for one example from a larger dataset of 210 stimuli). Every item and background image were taken from the COCO (Lin et al., 2014) or ImageNet (Deng et al., 2009) datasets, and appeared only one time across the experiment. A total of 210 images were presented. The stimuli were constructed following scene-syntax principles, i.e., without violations of support, interposition, size, or spatial plausibility, as detailed in (Ruarte et al., 2025). A bounding box (maximum of 80 × 80 pixels) was defined around each item. The full stimulus set is publicly available at https://osf.io/e97ws/.

#### Head digitization

Before the MEG acquisition, three electromagnetic coils were placed at three fiducial points on the participant’s head (nasion, left and right pre-auricular points), and digitization of these fiducial points and the head shape of the participant was carried out using a 3-D digitizer camera mounted on an iPad (Scanner app). Participants’ hair was tightly tied when possible to allow accurate surface capture. The information extracted with the camera does not only include the scalp surface but also part of the facial geometry, providing extra information to match with the surface from the individual’s MRI surface. This method was chosen over the traditional Polhemus digitization as it offers a much simpler and faster procedure while still providing sufficient head-shape information for accurate alignment. During MEG data acquisition, the location of the participant’s head within the MEG helmet was measured by energizing these coils. The location of the MEG sensors was co-registered to the brain anatomy by matching the digitized head surface to the head surface extracted from the anatomical MR image following (Brookes et al., 2011).

#### MEG recording

During the experiment, brain activity was recorded using a CTF 275 MEG, inside a magnetically shielded room. The experiment was presented to the participants through a projector, projecting an image onto a screen in the magnetically shielded room. On each trial, triggers were sent from the experiment display PC to the eye-tracker and MEG, indicating the beginning of the trial and the beginning and end of the search screen. The CTF system was configured to record at 1200 Hz and to apply 3rd order spatial gradient correction on the data to minimize the influence of environmental magnetic noise. MEG recordings of the empty room were performed regularly to use as background noise for source estimation.

#### ET recording

A MEG-compatible Eye tracker (ET; Eyelink 1,000 Plus, SR Research, Ontario, Canada) was used to record eye movements at a sampling rate of 1,000 Hz. The eye tracker was set to monocular mode to record the participant’s dominant eye and placed beneath the projector screen at approximately 68 cm from the participants’ eyes. The eye-tracker 9 pt. calibration took place at the beginning of the first block and was recalibrated between blocks when necessary. Calibration errors were kept below 0.5 deg. throughout the experiment. The ET data were stored in the Eyelink host PC and also converted to analogue signals using Eyelink’s Digital to Analogue converter. These signals were recorded as analogue sensors alongside the MEG data, allowing for precise detection of eye movement events (see ‘ET signal pre-processing’ section below).

#### MRI acquisition

For 14 of the 21 participants, a structural MRI was collected using a standard sMPRAGE T1 sequence in a 3 T Siemens scanner. The scanning parameters were as follows: TR = 2,000 ms, TE = 2.01 ms, TI = 880 ms, flip angle = 8 degrees, FOV = 256 × 256 × 160 mm, 1 mm isotropic voxel. For the remaining participants, the MNI 305 template brain was used in the later source modeling analysis.

### Pre-processing

#### ET signal pre-processing

The ET data was taken from the analogue sensors on the MEG to facilitate the synchronization with the MEG gradiometers’ data. The ET data was first rescaled from Volts to pixels following the Eyelink manual, then blinks were detected and removed by defining them as missing values in the signal, and then saccades and fixations longer than 50 ms were detected using Remodnav (Dar et al., 2021), an implementation of the Nystrom and Holmqvist’s velocity-based eye movement detection algorithm (Nyström and Holmqvist, 2010; see Supplementary Text 1 for details on the parameters used for the detection). Fixations on encoding and visual search screens were classified as fixations on targets, distractors, or ‘none’, depending on their distance to the items center using a 70 pixel (1.13 deg) radius threshold.

Finally, to determine if a delay was introduced by the Digital-Analogue Converter (DAC), epochs were time-locked to saccade onset, and the evoked response aligned to this onset was computed for the Grand Average of participants (GA). The evoked response presented a first peak approximately 10 ms before the onset of the saccades. This peak is known to take place right after saccade onset (Carl et al., 2012), so a − 10 ms shift was introduced in the ET data to compensate by removing the first 12 samples (sampling rate = 1,200 Hz) and padding the last 12 samples with empty values. After determining the DAC delay and compensating for it, the detection of fixations and saccades was re-run with the same parameters to correct the timing of the ocular events.

#### MEG signal pre-processing

The processing and analysis in this work were performed using MNE-python (Gramfort et al., 2013), following recommendations of well known analysis pipelines, such as FLUX (Ferrante et al., 2022). First, a 1 Hz bandwidth notch filter was applied to filter line noise at 50 Hz, 100 Hz, and 150 Hz. Bad sensors were manually selected based on visual inspection for each participant, for an average of 2.3 (SD = 3.3) bad sensors per participant. These sensors were Interpolated from neighboring sensors using minimum-norm estimation. Afterwards, noisy intervals on the signal were identified and labeled as “bad,” by visual inspection, and muscular artifacts were labeled using an automatic method from MNE package v. 1.5.1 (*annotate_muscle_zscore*) on high frequency filtered data (110–140 Hz). These intervals were excluded from all analyses reported in this work.

An Independent Component Analysis (ICA) was applied to every subject separately to remove ocular, muscular, cardiac, and noisy components. To this end, a copy of the preprocessed MEG signal was first downsampled at 200 Hz and filtered between 1 and 40 Hz, using 64 ICA components. The spectrum, topography, epochs (−50 to 100 ms) and evoked signal around saccades with left direction were plotted for each component individually and analyzed to determine if that component contained electrical noise, cardiac artifacts, and ocular artifacts. Since eye movements are present throughout the experiment, we followed the procedure introduced in (Plöchl et al., 2012) to identify eye-related components. In brief, this approach compares the variance around saccades with the variance around fixations and sets a semi-automatic threshold of 1.1 (higher variance associated with saccades) to define eye-related components. Using this approach, we identified approximately three to four ocular components corresponding to saccadic eye movements and blinks. In addition, approximately two components were identified as reflecting cardiac activity. The remaining rejected components were identified based on irregular or highly focal topographic patterns, and/or the power spectrum not showing the expected 1/f drop-off at high frequencies. These components reflected segments of high signal variance or minor SQUID jumps on individual channels that were too small to be detected by automated screening processes. Overall, an average of 11.6 (SD: 3.2) components per participant were removed. Once the artifactual components for each subject were defined, they were removed from the original unfiltered data and saved.

After ET signal processing and synchronization, events were marked on the MEG data, indicating each screen onset, and fixations, saccades, and button responses, to epoch the MEG data around times and conditions of interest.

#### Source estimation

Source estimates were computed using LCMV beamformer spatial filters. To this end, first, the head model of each participant was defined using its individual anatomical MRI, co-registered with the MEG data through the fiducial markers from the head digitization during MEG recording preparations following the procedure from Pan et al. (2023). The MRI data was processed and segmented using FreeSurfer to extract the Boundary Element Method (BEM) by assigning each voxel of the MRI data to a tissue class. For the 7 participants lacking an MRI scan, the MNI 305 template was used as the head model.

The BEM was later used to construct two source models for each participant: a volume model using a 5 mm-spaced isotropic grid and a surface model using ico-4 spacing of 2,562 sources per hemisphere. These models were then used to define forward models for volume and surface estimates.

The LCMV filters were constructed using the covariance matrices of the empty room recordings (1 min long), and the covariance matrices of the whole MEG recording for each participant. The covariance matrix was computed from the full raw data, excluding segments manually labeled as “bad segments,” or identified by the annotat_muscle_zscore function. This was done by splitting the data into 2 s epochs and excluding epochs which overlap with a segment marked as “bad”, then computing the covariance. These matrices were subject to regularization of 0.05 (5% of the sensor power) before inverting them in the beamformer calculation. Using all the MEG recordings provides a robust estimation of the covariance matrix and also avoids the need for biased LCMV filters for each condition from which sources would be estimated. To construct spatial filters for specific frequency bands (theta: 4–8 Hz, alpha: 8–12 Hz, beta: 12–30 Hz), the sensor data was first filtered with a 4th order Butterworth IIR filter, and source estimates were computed from the filtered sensor data (Barratt et al., 2018). The empty room recordings were subject to the same pre-processing and artifact removal methods as the MEG recording before being used in the LCMV filter. At each source location (defined by surface or volume source models), dipole activity was estimated in three orthogonal directions using a vector beamformer. Then, the scalar amplitude was computed as the norm of that vector, representing the total source power independent of orientation. These LCMV filters were finally applied to epoched or evoked time series and covariance matrices of epoched data. The results in individual participants’ volume source space were then morphed onto a common space, in this case, the MNI 305 template, where homologous points across participants were located at the same location, allowing for the signals and power estimates in source space to be directly averaged across participants.

Surface source estimates were divided into 148 discrete cortical regions, defined based on the Destrieux atlas (Destrieux et al., 2010). Singular value decomposition was applied to the time courses within each label, and the first right-singular vector was taken as the representative label time course. This yielded one signal per cortical region, from which time-frequency power was computed as detailed below.

### MEG analysis

#### Time-frequency analysis

The time-frequency analysis for real and virtual sensors was performed using Morlet wavelet filters in 1 Hz steps with MNE (Gramfort et al., 2013). For each frequency, the number of cycles was set as frequency / 2, which resulted in a constant temporal resolution across frequencies (corresponding to a fixed wavelet duration of 0.5 s), while providing increased spectral resolution for higher frequencies. This choice balanced the temporal and spectral resolutions for the analyses.

#### Deconvolution analysis

To accurately isolate and characterize the neural responses associated with individual eye movement events in our free-viewing paradigm, a deconvolution analysis was employed. This approach is crucial for disentangling overlapping neural signals that arise from the rapid succession of fixations and saccades inherent to natural visual exploration.

The continuous MEG signal was modeled as a linear summation of responses to discrete events (e.g., fixation onsets, saccade onsets), where each event is associated with a specific neural response kernel. Specifically, methods based on the Temporal Response Function (TRF) approach (Lalor et al., 2006; Dimigen and Ehinger, 2021; Care et al., 2023; Gonzalez et al., 2024) were used. TRFs characterize the linear mapping between a continuous stimulus (or, in our case, a series of discrete events modeled as impulses) and the continuous MEG signal. Each event of interest (e.g., target fixation, distractor fixation, saccades, and button presses), and its characteristics (such as fixation rank, duration, or MSS) was represented as an impulse at its onset time within a continuous regressor. Using MNE’s Receptive Field toolbox, a linear model was then constructed in which the MEG signal was predicted by convolving these event regressors with their respective TRFs. These TRFs themselves are essentially time-varying weights that capture how the brain responds over time following each event. Estimation of the TRFs was performed by minimizing the error between the predicted and observed MEG signals, with Ridge regularization (alpha = 1,000) to prevent overfitting. This process effectively “unfolds” the superimposed neural responses.

#### Connectivity analysis

To investigate the amplitude coupling between different brain regions, a connectivity analysis was performed using amplitude envelope correlation (AEC) with pairwise orthogonalization for leakage correction (Sareen et al., 2021; Brookes et al., 2012). Surface sources were estimated from the epoched MEG data of each participant corresponding to the fixations to the target and distractors using an LCMV Beamformer filter and aggregated into regions (following the procedure described above). The envelope of the signal for each region was extracted, and the correlation between each pair of time-series was computed, using pairwise orthogonalization for leakage correction (Boto et al., 2021; Rier et al., 2024). This method is robust against spurious synchronizations due to spatial leakage (Ragazzoni et al., 2019). However, it is worth noting that amplitude envelope correlation can partially reflect shared evoked structure when applied to task data with prominent components, Therefore, AEC connectivity reflects coordinated amplitude modulations across regions, rather than pure oscillatory synchronization. The resulting adjacency matrix was standardized and then averaged across participants when presenting grand average results. For comparing the connectivity of two conditions, the connectivity matrices of each participant were subtracted without any normalization or standardization, obtaining one difference connectivity matrix per participant. That matrix was then standardized by estimating z-scores for each participant and averaged across participants to get the difference in connectivity for the average of subjects.

To represent the connectivity of brain regions we use three visualization methods: Connectome plots, that show nodes located in the brain, and the links between those nodes, brain surface plots showing the connectivity degree of each region, and the connectivity matrix itself. The connectome plots present the strongest connections in the connectivity matrix. This is achieved by setting a threshold as the 150th highest absolute value of the matrix. Links with strictly higher absolute values than that threshold are kept, resulting in 149 links at most depending on repeated values in the matrix. The connectivity degree plot shows the sum of the connectivity weights of each node or region (connectivity degree for continuous valued networks).

To assess the significance of the difference in connectivity between hemispheres, the average connectivity matrix was used to select the 150 highest links in absolute value. Those links were extracted from the connectivity matrix of each subject and split into left and right hemispheres and used in the analysis. Connectivity values were averaged for each participant and hemisphere separately, yielding a left and right connectivity index for each participant. Finally, the left and right connectivity values were subject to a Wilcoxon signed-rank test.

#### Cluster statistics

The statistical significance of the results (both in time-frequency space or power source space and virtual sensor signals) was assessed by nonparametric cluster-based permutation tests implemented in MNE-python (Maris and Oostenveld, 2007; Gramfort et al., 2013). For time-frequency sensor-space data, the clustering was estimated on 3 dimensions (time x frequency x sensors) on the basis of spatial, spectral, and temporal adjacency. For time-frequency source-space data, surface source estimates were aggregated into regions (see Source estimation) and the clustering was estimated on 2 dimensions for each region (time x frequency) on the basis of spectral, and temporal adjacency. Source-space data statistics were performed on volume source estimates, both for source space power or virtual electrode time series. For source-space time series data, clustering was based on both spatial and temporal adjacency. Finally, for average power data in source-space, clusters were computed considering only spatial adjacency. The adjacency matrix was defined using MNE (*spatial_src_adjacency*) function, which takes the volume source model as input and returns the adjacency matrix to be used in the cluster permutation test.

Mass-univariate comparisons were thresholded using a t-value of 2.84 (corresponding to *p* = 0.01, d.f. = 20) to define candidate clusters. For each subject, the difference between conditions (task vs. baseline, or high vs. low memory load) was computed individually, and submitted to a one-sample permutation test, with 5,120 permutations. Clusters with permutation-based *p*-values smaller than 0.05 were considered statistically significant.

## Results

### Behavior and eye movements

**Figure 2:**
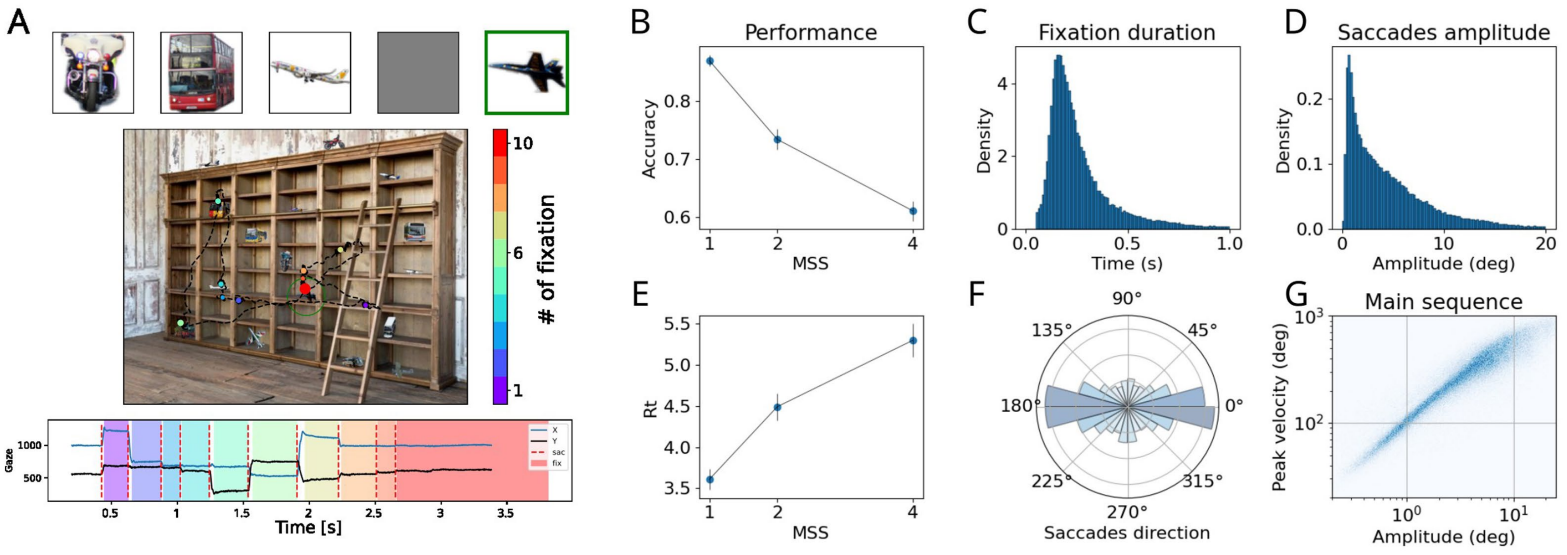
Behavioral performance and eye movement statistics during visual search. **(A)** Example trial for a memory set size MSS of 4. The top panel shows the memory set; the middle panel presents the scanpath over the visual search (VS) display with saccades as dotted black lines and fixations as colored dots indicating their temporal order (colorbar); the bottom panel plots horizontal (X) and vertical (Y) gaze positions over time (measured from visual search screen onset). **(B)** Mean accuracy (+-S. E. M.) across different memory set sizes. **(C)** Distribution of fixation durations during visual search. **(D)** Saccade amplitude distribution. **(E)** Mean reaction times (+-S. E. M.) across different memory set sizes. **(F)** Saccade angular distribution. **(G)** Main sequence plot showing the relationship between saccade amplitude and peak velocity during visual search. Images were adapted from the COCO dataset (Lin et al., 2014; https://cocodataset.org/, CC BY 4.0) and ImageNet (Deng et al., 2009; http://www.image-net.org/, non‑commercial research license).

Participants were asked to memorize a set of items of different sizes (MSS = 1, 2, 4) and then search for any of them in a natural image including several items (Figure 2A). Behavioral performance showed an accuracy decrease with memory set size (Figure 2B), and a characteristic logarithmic increase of response times with memory set size (Figure 2E; Wolfe, 2012; Barbosa et al., 2024). Eye movements analysis for fixation durations, saccade amplitude and direction, and the main sequence of saccades (Figures 2C,D,F,G) are consistent with established eye movements patterns across tasks (Otero-Millan et al., 2008). Supplementary Figure S1 contains the information regarding the distributions of fixation duration, saccade amplitude and the number of fixations per trial, separately for each MSS.

### Task-related brain activity

**Figure 3:**
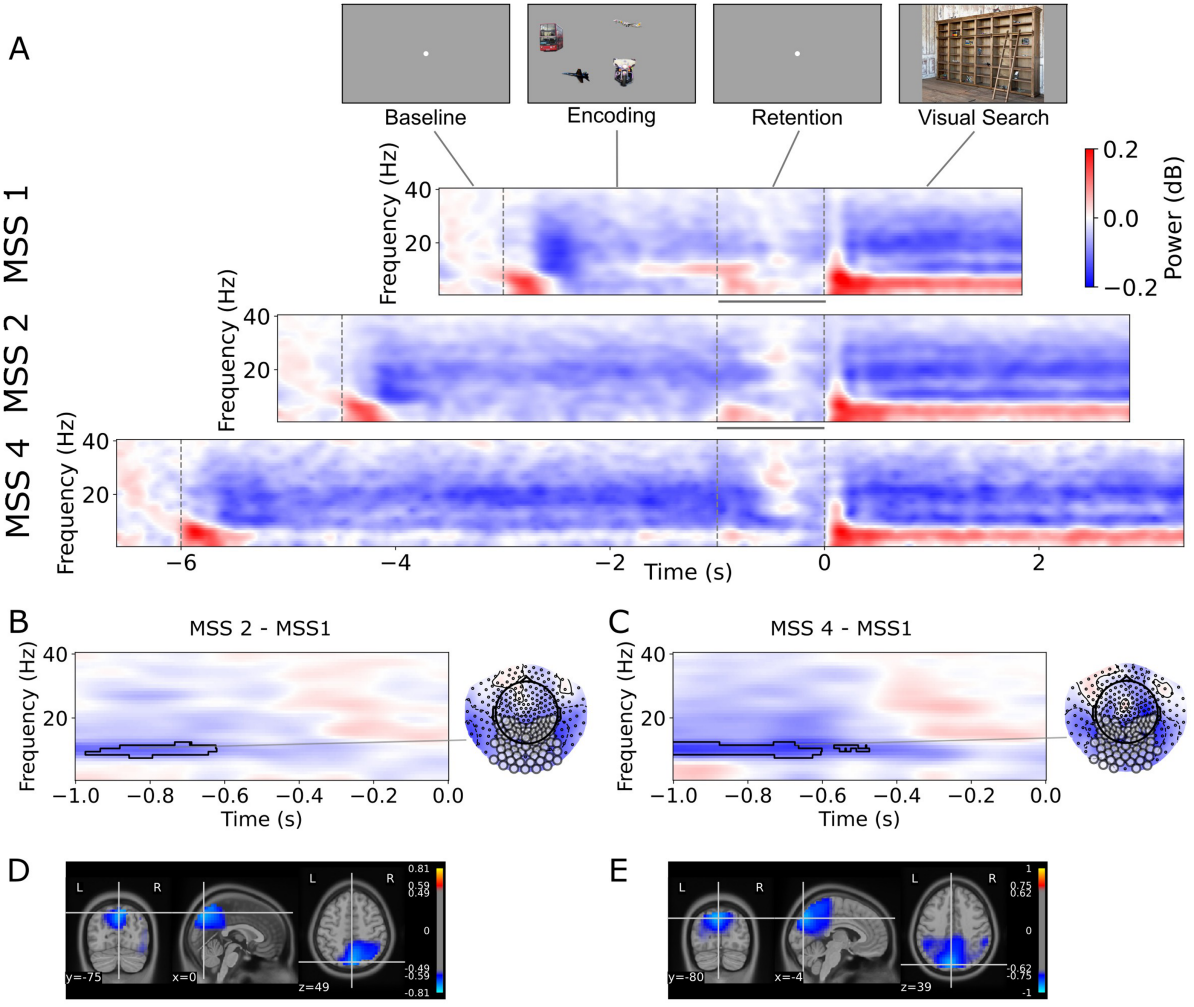
Time-frequency spectrograms (TFS) and memory load effects. **(A)** Schematic of the task (top panel) and TFSs across memory set sizes (middle panels) from parietal and occipital sensors. **(B,C)** Difference in spectral power for medium/high vs. low memory set size during the retention period, shown in sensor space. Significant clusters, delimited by black contour lines, were estimated with a permutations test (see Methods). These clusters reflect time-frequency points where at least half of the sensors of the region were significant (*p* < 0.05), with *p*-values of 0.0002 in both cases (MSS 4 - MSS1 and MSS 2- MSS 1). **(D,E)** Difference in spectral power for medium/high vs. low memory set size during the retention period, shown in source space. Source space voxels with significant power differences between memory set sizes show maximum differences in the superior parietal lobe (Brodmann 7) in both cases. Images were adapted from the COCO dataset (Lin et al., 2014; https://cocodataset.org/, CC BY 4.0) and ImageNet (Deng et al., 2009; http://www.image-net.org/, non‑commercial research license).

Figure 3 shows an overview of the power changes over the parietal and occipital sensors throughout the task across all memory loads. In the encoding phase, oscillatory power increased in the theta band (4–8 Hz) approximately 100 to 200 ms after screen onset and then decreased strongly around 500 ms in the alpha (8–12 Hz) and beta band (12–30 Hz). In the retention interval, alpha power significantly decreased with memory load, both in sensor space and in source space.

To compare the activity over the retention period, a cluster-based repeated measures ANOVA across memory load conditions was performed, which revealed two significant clusters (Supplementary Figure S2A). The first large cluster was found in the alpha band, extending partially to low beta, spanning up to approximately 0.65 s into the retention period (*p* = 0.0002). A second smaller cluster in the beta band, extending until approximately 0.25 s in retention period, was also found. Given the significant differences in alpha band activity across memory loads, this analysis was followed by pairwise comparisons. Figures 3B,C show significant differences (*p* < 0.0002, nonparametric cluster-based permutation test, see Methods) in alpha power between medium (MSS = 2) and low (MSS = 1) memory load conditions, as well as between high (MSS = 4) and low (MSS = 1) conditions. In contrast, there were no significant differences between high and medium memory load conditions. These results support alpha power suppression in posterior regions as a reliable marker of WM load, with WM reaching its maximum capacity for encoding natural objects at medium memory load. Source-level power during the retention period was estimated using an LCMV Beamformer applied to covariance matrices derived from the corresponding epochs, filtered in the alpha band (see Methods Section). To study the effect of memory load in source space, the difference between the source power for each load condition was computed, and a permutations test was performed on source space power estimates. This resulted in significant differences between high vs. low memory load (*p* = 0.0029) and medium vs. low memory load (*p* = 0.0039) conditions (Figures 3D,E). In both cases, the largest significant differences were found in the superior parietal lobe (Brodmann 7). No significant differences were found for high vs. medium memory load conditions. However, the decrease in alpha power with memory load was not specific to the retention period. During memory encoding, we found significant differences in the alpha parietal power between high (MSS = 4) and medium (MSS = 2) when compared to low (MSS = 1) memory load conditions (see Supplementary Figure S2).

### Memory formation: task and fixation-related responses

For the high memory load condition, where there was a sufficiently large and balanced number of correct and incorrect responses, we investigated whether differences in amplitude or synchronization of oscillations were predictive of successful memory performance. These analyses were conducted separately for power, phase synchronization, on fixation, and saccade events. However, no significant differences in power nor phase synchronization (estimated using inter-trial phase coherence) were found when comparing correct and incorrect trials (Supplementary Figure S3). This analysis was performed both on a “whole trial” basis, with the power and phase during the encoding period, and also based on relevant events, aligning the data to individual saccades to items, since previous studies have reported stronger alignment of oscillations with saccade onset rather than fixation onset (Pan et al., 2023).

Next, we conducted a separate deconvolution power analysis for the alpha and beta bands, the two frequency ranges connected to our hypothesis H2. In sensor space, the deconvolution analysis shows significant effects of subsequent correct target identification in both frequency bands, with the maximum number of significant sensors occurring at approximately 290 ms for alpha and 250 ms for beta oscillations. We then performed a source level analysis, by extracting time-frequency power plots for each cortical region. This identified only one small region (right anterior occipital sulcus) showing a significant cluster in the beta range.

### Effect of memory load on fixation-related responses

**Figure 4:**
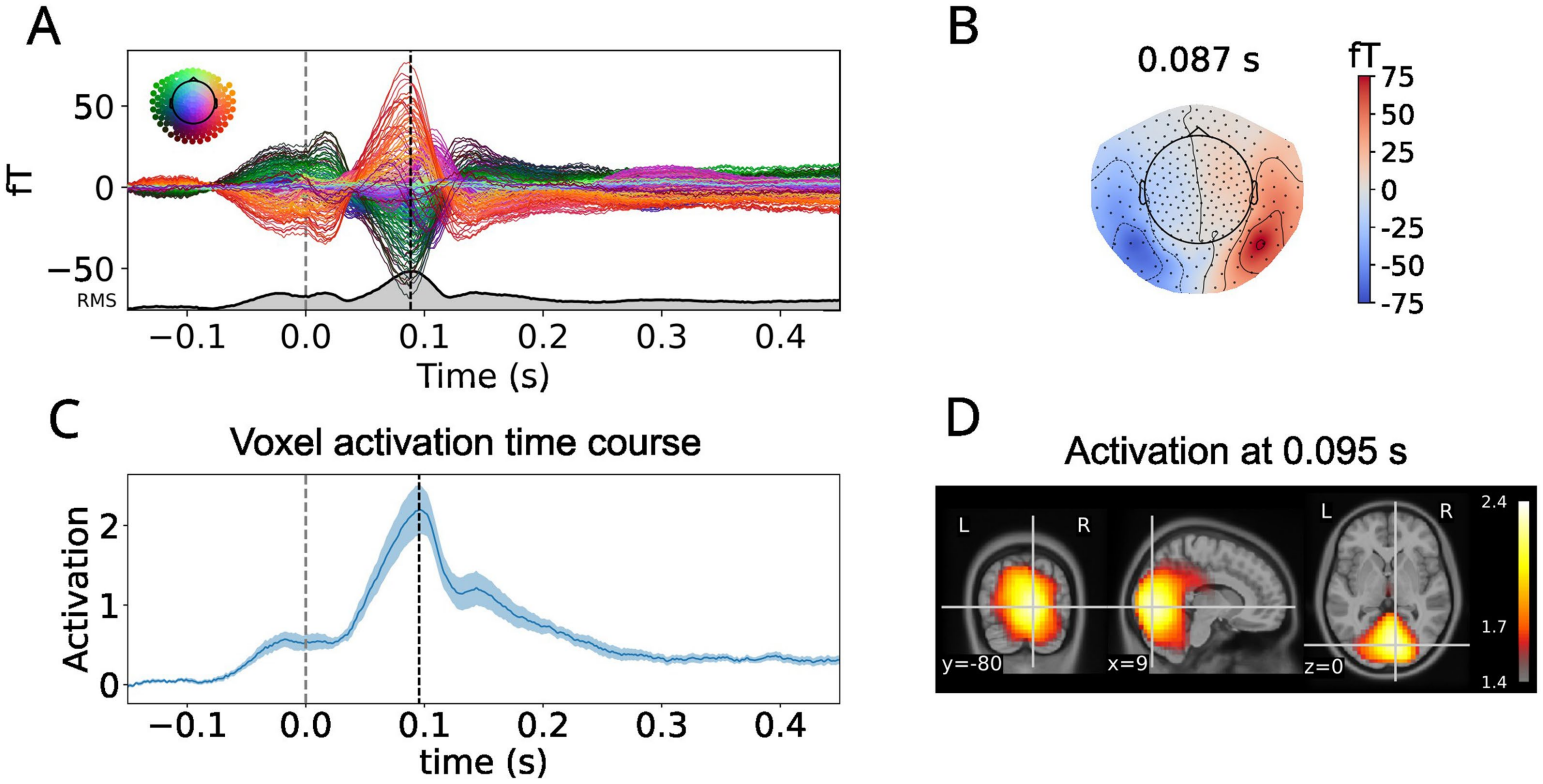
Lambda wave response. **(A)** Sensor-level evoked responses to fixations on items and targets in the visual search task, aligned to fixation onset (t = 0). The response presents a peak around 87 ms post-fixation. **(B)** Topographic plot of sensor evoked response at 87 ms post-fixation. **(C)** Virtual electrode time series from a 0.125 cm3 region centered on the occipital cortex (MNI: [9, −80, 0]). The time series represents the average activation to fixations on targets and distractors, aligned to fixation onset (t = 0). The time course presents peak activation at 95 ms. **(D)** Source-level estimates showing activation at 95 ms, localized to V1 (MNI: [9, −80, 0]). Images were adapted from the COCO dataset (Lin et al., 2014; https://cocodataset.org/, CC BY 4.0) and ImageNet (Deng et al., 2009; http://www.image-net.org/, non‑commercial research license).

Before investigating the effect of memory load, the early visually evoked component in free viewing, the lambda response (Kazai and Yagi, 2003), was characterized. Sensor and source-level fixation-related responses were computed for all fixations to items in the visual search screen. Figure 4A shows the evoked response across all sensors, presenting a peak around 87 ms after fixation onset. The topography at that time is shown in Figure 4B, where the occipital sensors present the highest activity, with a positive activation to the right and negative to the left. Figures 4C,D present the source activations for the fixation-related field after fixation onset. The voxel with highest activation (MNI: [9, −80, 0]) is located in the V1 region. The time course of the associated virtual sensor at this location presents a peak around 95 ms after fixation onset. These source-level results are consistent with the sensor level data, explaining the bilateral pattern observed in the occipital sensors as being generated by a dipole pointing from the posterior to the anterior pole within the visual cortex. This is consistent with previous concurrent EEG and eye movements studies (Kaunitz et al., 2014; Ries et al., 2016, 2018), in which a strong visually evoked lambda response emerges, with a peak approximately at 100 ms post-fixation.

**Figure 5:**
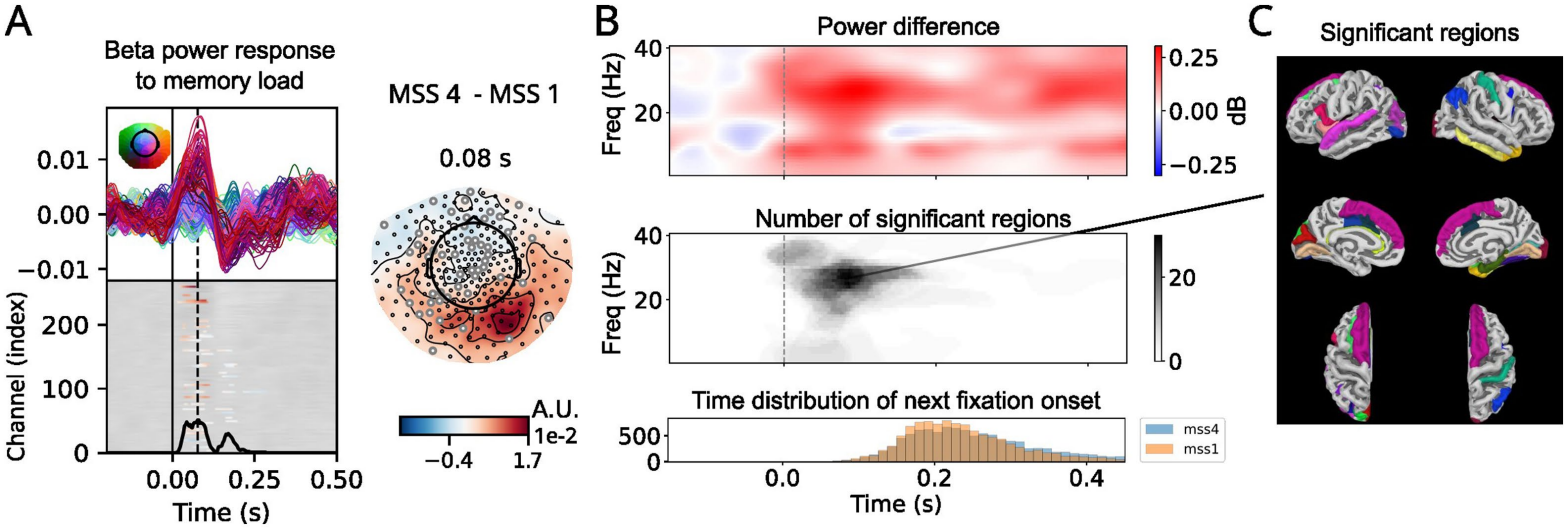
Fixation-related activation and modulation of power by working memory load. **(A)** Sensor-level deconvolution results. The top-left panel presents the temporal response function (TRF) corresponding to fixations on target and distractor items in high memory load and correct trials, averaged across participants for all sensors. The bottom-left panel shows the channel responses with non-significant time-points masked. The black trace indicates the aggregated number of significant sensors over time, presenting a maximum at approximately 80 ms. The right panel shows the sensor-level topography at the maximum time (80 ms), showing a strong occipito-temporal response. Significant sensors are denoted by white circles. **(B)** Power differences in distractor fixations during visual search in trials with high (MSS = 4) versus low (MSS = 1) memory load. The top panel illustrates time-frequency representations of power modulation, with one prominent cluster indicating significant differences across a large number of regions (middle panel). The bottom panel shows the distribution of the next fixation onset (i.e., current fixation duration + next saccade duration) for both conditions, confirming that observed differences arise during the current fixation. **(C)** Cortical regions presenting significant differences between MSS4 and MSS1 fixations on items. Images were adapted from the COCO dataset (Lin et al., 2014; https://cocodataset.org/, CC BY 4.0) and ImageNet (Deng et al., 2009; http://www.image-net.org/, non‑commercial research license).

To assess the effect of memory load on fixation-related responses in visual search, a deconvolution analysis based on Temporal Response Functions (TRF) was performed on the sensor-level power data of the beta band. Figure 5A shows the TRF to fixations on target and distractor items in correct trials of high memory load (MSS 4). This shows a peak of significant channels at 80 ms approximately, located to the parietal and occipital region. This indicates that high memory load elicits significant beta-band activation in the parietal and occipital regions, even after accounting for the temporal overlap. Supplementary Figure S4 shows the results for the entire deconvolution analysis, presenting the TRF for the remaining features (fixations, fixation rank, saccades, button presses).

To further validate these results, we estimated surface source responses for epochs aligned to the onset of distractor fixations both in MSS 4 and MSS 1 trials. We then aggregated those responses into cortical regions following the procedure mentioned in the Methods section, and finally computed the time-frequency response of each of those regions to MSS 4 and MSS 1 fixations. The contrast between fixations under high (MSS = 4) and low (MSS = 1) memory load is presented in Figure 5B. Significant activations were most prominently observed in the beta band at approximately 90 ms (*p* < 0.05, nonparametric cluster-based permutation test, see Methods). The brain regions showing these activations are shown in Figure 5C, extending from frontal to parieto-occipital areas.

To study if this effect was dependent on using fixation events, the analysis was repeated aligning epochs to saccade onset (Pan et al., 2023). This alternative approach gave similar results, both in terms of the number of regions presenting significant differences, as well as the effect of beta band oscillations on memory load (see Supplementary Figure S5).

### Target recognition

**Figure 6:**
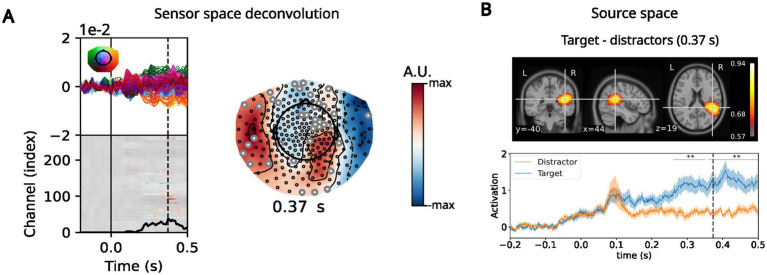
Evoked fixation-related responses to targets and distractors during visual search. **(A)** Sensor-level deconvolution analysis results. The top-left panel presents the TRF corresponding to fixations on target items in correct trials, averaged across participants for all sensors. The bottom-left panel shows the channel’s response masking non-significant time-points. The black trace indicates the aggregated number of significant sensors over time. The right panel shows the sensor-level topography at the time when the maximum number of significant sensors is found (370 ms), revealing a strong bilateral response in temporal sensors. Significant sensors are denoted by white circles. **(B)** Source-level differential activation between target and subsampled distractor fixations at 370 ms, with a maximum activation in the right inferior parietal lobe (MNI: [44, −40, 19]). Virtual electrode time series from 0.125 cm3 regions centered on these specified voxels reveal significant differences between target and distractor fixations from approximately 220 ms to 500 ms (*p* < 0.0015 for the temporo-parietal junction). Images were adapted from the COCO dataset (Lin et al., 2014; https://cocodataset.org/, CC BY 4.0) and ImageNet (Deng et al., 2009; http://www.image-net.org/, non‑commercial research license).

To analyze the effect of target recognition, a deconvolution analysis was performed. Figure 6A shows the TRF to target fixations, representing the difference in response between correct and incorrect trials. The time series in the top left panel represent the response of each sensor to target fixations in correct trials, showing a small peak at 100 ms in occipital sensors, followed by a broader peak from around 200 ms onwards in temporal sensors. The bottom panel also shows each sensor fitted signal, masked by the significance assessed by a permutations test with 5,120 permutations (*p* < 0.05). A black line indicates the total number of significant sensors at each time, showing a significant effect from around 200 to 450 ms (Sassenhagen and Draschkow, 2019). The maximum number of significant sensors is achieved at 370 ms. The right panel shows the topography of the weights of each sensor at 370 ms, with its largest values in the bilateral temporal (Herrmann and Mecklinger, 2000), and in right tempo-parietal sensors. Results for the full deconvolution model, including target fixations, saccades, fixation rank, and button presses are shown in Supplementary Figure S6.

Fixation-related fields for fixations on targets and distractors in the visual search phase were computed, and used to estimate source-level activations. These analyses included only fixations (for distractors and targets) from trials where the target was present, and responses were correct. Due to the imbalance between conditions (around 15:1 fixations on distractors vs. target), fixations to distractors were randomly subsampled for each participant. The source-level evoked responses were computed separately for each participant and condition (target vs. distractor). For visualization purposes, the data from each condition, as well as their difference, were averaged across participants to generate a Grand Average response for each condition, and for the difference map. The top panel of Figure 6B shows the Grand Average difference source activations map for the fixation-related field at 370 ms after fixation onset. The virtual electrode with maximum activation at that time-point is present around the tempo-parietal junction, specifically in the Right superior temporal gyrus (Brodmann area 22), although increased activation is visible across many areas. Statistical significance was assessed through a paired non-parametric cluster-based permutations test (*p* = 0.00097, t; Supplementary Figure S7). The bottom panel shows the Grand Average time courses of that virtual electrode for target and distractor conditions separately, with significant differences emerging from 220 ms onwards (*p* < 0.0015).

To further characterize the widespread activation observed during target detection, we performed a secondary supporting functional connectivity analysis during target and distractor fixations. Supplementary Figure S8 shows connectivity matrices in the alpha band and connectome plots for each condition, as well as their difference (target - distractors). Both target and distractor fixations show strong bilateral temporal-frontal synchronization. Consistent with the analysis of fixation-related fields (Figure 6B), the contrast between conditions shows a strong lateralization effect, with significantly higher connectivity for fixations on targets in the right hemisphere (Wilcoxon signed-rank test, *p* = 0.00000095). Notably, all participants showed stronger connectivity for distractor fixations on the left hemisphere, and stronger connections for target fixations on the right hemisphere (Supplementary Figure S8).

## Discussion

In this work, we show the contribution of brain oscillations and fixation-related activity to hybrid visual and memory search tasks. Memory-dependent effects were observed in parietal alpha power, with attenuation starting in the encoding phase and persisting throughout the retention interval. We also found memory load effects on fixation-related responses, particularly in the beta band, across a large number of frontal and parieto-occipital regions. Neural signatures of target detection during the visual search phase emerged from fixation-related responses, as seen in sensor space, source space, and functional connectivity analyses. The timing and topography of these effects align with findings from fixed-gaze neuroimaging studies (Herrmann and Mecklinger, 2000; Kok, 2001; Polich, 2007), free-viewing EEG (Kaunitz et al., 2014; Kamienkowski et al., 2018; Gordon et al., 2024), as well as intracranial and simultaneous EEG-fMRI recordings (Halgren et al., 1998; Bledowski et al., 2004).

Consistent with earlier findings reporting alpha/beta desynchronization during encoding (Fukuda et al., 2015; Griffiths et al., 2019), we found significant markers of such effect aligned to stimulus presentation. This started during the encoding phase, in line with previous fixed gaze working memory studies (Proskovec et al., 2019) and persisted during the retention period (Chen et al., 2022). The attenuation of posterior alpha oscillations was modulated by memory load, thus supporting our experimental hypothesis H1. Our findings are consistent with a large corpus of works reporting either an amplification of alpha oscillations linked to distractor inhibition, where alpha amplifications in non-relevant task regions are thought to reflect the suppression of distracting stimuli and facilitate more efficient communication between task-relevant regions, or an attenuation of alpha oscillations in task-relevant regions, where alpha oscillations are thought to serve a genuine mnemonic function (Jensen and Mazaheri, 2010; Bauer et al., 2014; Erickson et al., 2019). Given the involvement of posterior regions during encoding and retention of fine-grained perceptual representations, as opposed to simple stimuli, our results support the role of posterior alpha oscillations and the maintenance of working memory representations (van Ede, 2018).

Contrary to our second hypothesis (H2), we did not find significant differences in phase synchronization or overall oscillatory activity when comparing trials in which the target was found or not. As reviewed by (Hanslmayr and Staudigl, 2014), evidence from noninvasive M-EEG studies and intracranial EEG during the encoding of memories typically shows changes in amplitude or phase synchronization linked to successful memory formation. These changes manifest as increases or decreases in theta and alpha frequency bands, and as decreases in the beta band. They are also dependent on the way in which information is processed during encoding, with alpha and beta power decreases predicting memory formation only in semantic tasks (Hanslmayr and Staudigl, 2014). The underlying hypothesis is that successfully encoded memory traces are reinstated during retrieval. To further examine this question, we conducted an additional deconvolution analysis focused on the alpha and beta bands. This analysis revealed some evidence for differences linked to subsequent target identification. However, the effect was confined to only one source region in the boundary between the temporal and occipital lobes. As such, although this analysis provides preliminary support for this effect, results should be interpreted with caution. There are also a number of possible reasons why we did not observe consistent differences in our experiment. First, we only conducted this analysis for the high memory load condition, as there was a larger imbalance between correct and incorrect trials for the other set sizes, because of the higher performance. Second, while memory retrieval is usually assessed directly in memory experiments, in our experiment, successful trials corresponded to targets in which the target was found, thus leaving the possibility that the target was presented but not fixated on during the visual search process. Third, with the exception of Staudigl et al. (2017), most of the existing evidence comes from experiments conducted under fixed gaze in which, arguably, differences between networks involved in successful and unsuccessful encoding might be easier to detect. In sum, future studies with designs optimized for subsequent memory contrasts may help to clarify the robustness and relevance of these effects.

We observed effects of memory load on fixation-related responses, consistent with our hypothesis H3. When participants attempted to recognize a target among a series of memorized items, beta oscillations increased with memory load around 100 ms post-fixation in a large number of frontal and parietal regions. Frontal beta oscillations have traditionally been associated with top-down control mechanisms (Miller et al., 2018), task demand and attentional effort (Gross et al., 2004; Donner et al., 2007; Siegel et al., 2012; Stoll et al., 2016). During a free-viewing visual search task, Pesaran and colleagues found that beta coherence was stronger during free-choice compared to instructed decisions, suggesting that top-down signals from frontal regions shape decision dynamics (Pesaran et al., 2008), see also Bichot et al. (2005). In another experiment in monkeys performing a test of cognitive control, Stoll and colleagues found a significant increase in beta oscillations only when cognitive control was required (Stoll et al., 2016). Our findings support that the beta modulation in temporal and frontal regions is related to early access to working memory, associated with the recognition attempt, which becomes more pronounced under higher memory demands.

In line with our hypothesis H4, we found robust neural markers of target detection. The neural mechanisms underlying target detection in visual search have been extensively studied using electrophysiological and neuroimaging approaches. One well-established neural signature of target detection is the P3 component (and its magnetic counterpart, P3m), which emerges around 300–600 ms after stimulus onset and has traditionally been linked to contextual processing of target-related information (Polich, 2007). The P3m topography we observed, with a maximum over temporal sensors, is consistent with previous fixed gaze works (Herrmann and Knight, 2001), and generalizes previous findings of concurrent EEG and eye tracking recordings during free viewing visual search (Kaunitz et al., 2014; Touryan et al., 2017; Kamienkowski et al., 2018). Importantly, the target-related effect remained significant even when a deconvolution approach was used. Our results show a widespread network involved in target processing. Intracranial recordings from neurosurgical patients have identified P3 generators in widespread regions, including the hippocampus, superior temporal sulcus, ventrolateral prefrontal cortex, and intraparietal sulcus (Halgren et al., 1998). By applying source reconstruction techniques for MEG recordings, we found maximum activation in the right inferior parietal lobe. Bledowski and collaborators used a three-stimulus oddball task with combined EEG and fMRI recordings and reported activation of a widespread cortical network, with pronounced activation in parietal regions including the inferior parietal lobe and the posterior parietal cortex, as well as a strong lateralized effect with greater activation associated to targets in the right hemisphere (Bledowski et al., 2004). This is also consistent with the results of our functional connectivity analysis for fixations on targets and distractors, which revealed increased brain connectivity in the right hemisphere for targets, while the connectivity in the left hemisphere showed larger connectivity for fixations on distractors. These findings, however, should be interpreted in light of several methodological and conceptual limitations.

First, it is worth noting that covariance-based spatial filtering may suppress highly correlated (e.g. bilateral) sources, potentially biasing apparent lateralization, and this limitation is not fully mitigated by analyses of power changes or by leakage correction. Second, while traditional neuroimaging experiments normally present carefully designed isolated stimuli on blank backgrounds, we have digitally inserted target and distractor items within structured real-world scenes. Our paradigm does not capture many aspects of real-world behavior, such as motion, viewpoint shifts, occlusion, or embodiment. However, our aim was not to recreate fully naturalistic search, but to achieve a balance between ecological and internal validity. The resulting stimuli preserve key aspects of real-world scene structure while still allowing experimental control. This strategy is consistent with recent perspectives calling for research programs that better reflect the actual problems the brain confronts in everyday settings (e.g., Breakspear, 2017; Nastase et al., 2020), sharing the view that using naturalistic stimuli is advantageous to better approximate how the brain functions ‘in the wild’ (Mobbs et al., 2018; Sonkusare et al., 2019). However, our paradigm remains less immersive than head-mounted or fully dynamic real-world search environments, and should be interpreted as a controlled free-viewing paradigm with natural images rather than an “in the wild” paradigm. Future work could extend these approaches towards richer settings. Second, unlike fixed-gaze experiments that suppress natural eye movements, our task encouraged participants to perform as many saccadic eye movements as needed (i.e., free-viewing search). This introduces analysis challenges, but we built on extensive previous experience from concurrent M-EEG and eye movements recordings (Carl et al., 2012; Plöchl et al., 2012; Kaunitz et al., 2014) to identify and correct for artifactual components. Although we cannot rule out the contamination of eye movements in the processed data, there is good evidence to support the robustness of the fixation-related approach, including the identification of a robust lambda wave component. This component, estimated from co-registered MEG and MRI signals, localized to V1 matching the localization reported by a previous EEG study (Kazai and Yagi, 2003). Third, even if the muscular artifacts are perfectly accounted for, the processing of information during each fixation in natural vision will likely be influenced by the activity associated with the previous fixation. This issue was addressed by applying a deconvolution method (Lalor et al., 2006; Litvak et al., 2013; Crosse et al., 2016; Care et al., 2023), which has been shown to robustly handle unconstrained scenarios and provide more precise estimates of fixation-related neural activity. Future work should extend deconvolution methods to further explore the neural mechanisms underlying visual search, focusing on fixation- and saccade-level representations, examining how these processes depend on oscillatory activity, and integrate deconvolution with high-resolution MEG source reconstruction. Finally, it is worth noting that covariance-based spatial filtering may suppress highly correlated (e.g., bilateral) sources, potentially biasing apparent lateralization, and this limitation is not fully mitigated by analyses of power changes or by leakage correction.

In conclusion, our study sheds light on the neural mechanisms of visual search under context-rich free viewing conditions, emphasizing the role of alpha and beta oscillations, and fixation-related responses in guiding target detection and working memory processes. By bridging the gap between traditional laboratory studies and real-world scenarios, our findings contribute to a deeper understanding of visual cognition and its neural underpinnings.

## Data Availability

The raw data supporting the conclusions of this article will be made available by the authors upon reasonable request.
