# Supplementary material for: Mapping neural activity during naturalistic visual and memory search
Source: Front Hum Neurosci. 2026 Jul 9;20:1812919. doi: 10.3389/fnhum.2026.1812919 (PMC13391513; doi:10.3389/fnhum.2026.1812919)
Supplement: Supplementary file 1 [file Data_Sheet_1.docx]

Supplemental Material

# Supplementary Data

## Supplementary Text 1: Eye movements detection

REMoDNaV (Dar et al., 2021), with the implementation found here: <https://github.com/psychoinformatics-de/remodnav> was used to detect eye movement events.

Parameters used for the detection:

--savgol-length: 0.0195

--min-pursuit-duration: 2 (To avoid getting smooth pursuit events)

--max-pso-duration: 0.0 (To avoid having Post saccadic oscillation events)

--min-fixation-duration: 0.05 (Min fixation duration 50 ms)

--max-vel: 5000

Additionally, saccades with peak velocity over 1500°/s and fixations with an amplitude over 1.5° were removed.

# Supplementary Figures

## Supplementary Figure S1: Eye movements for MSS


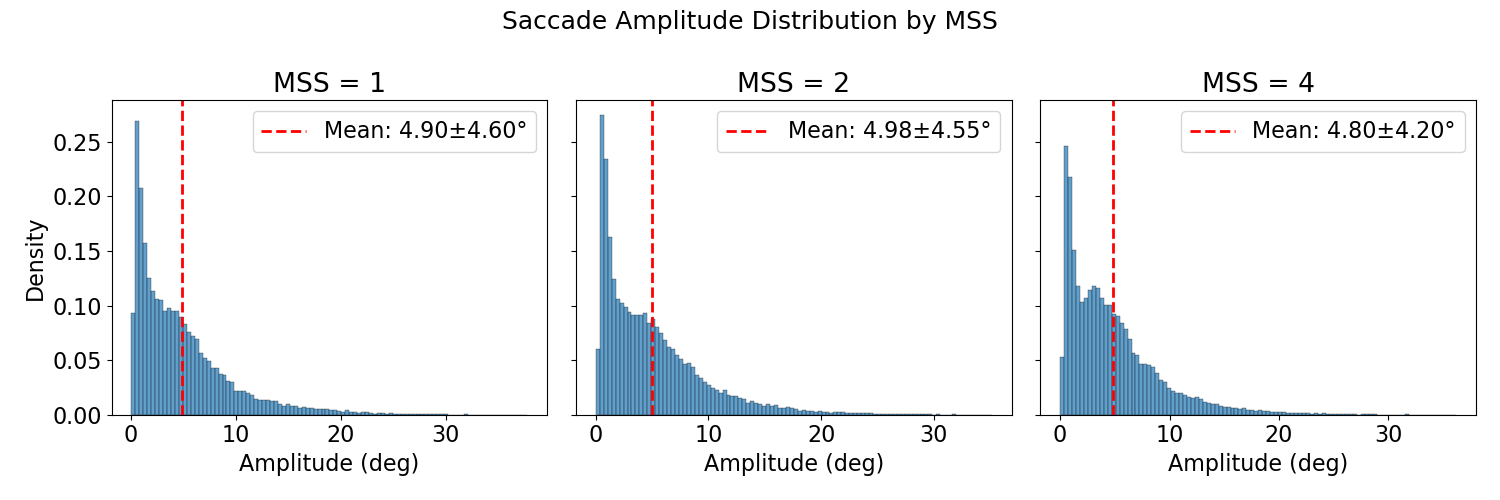

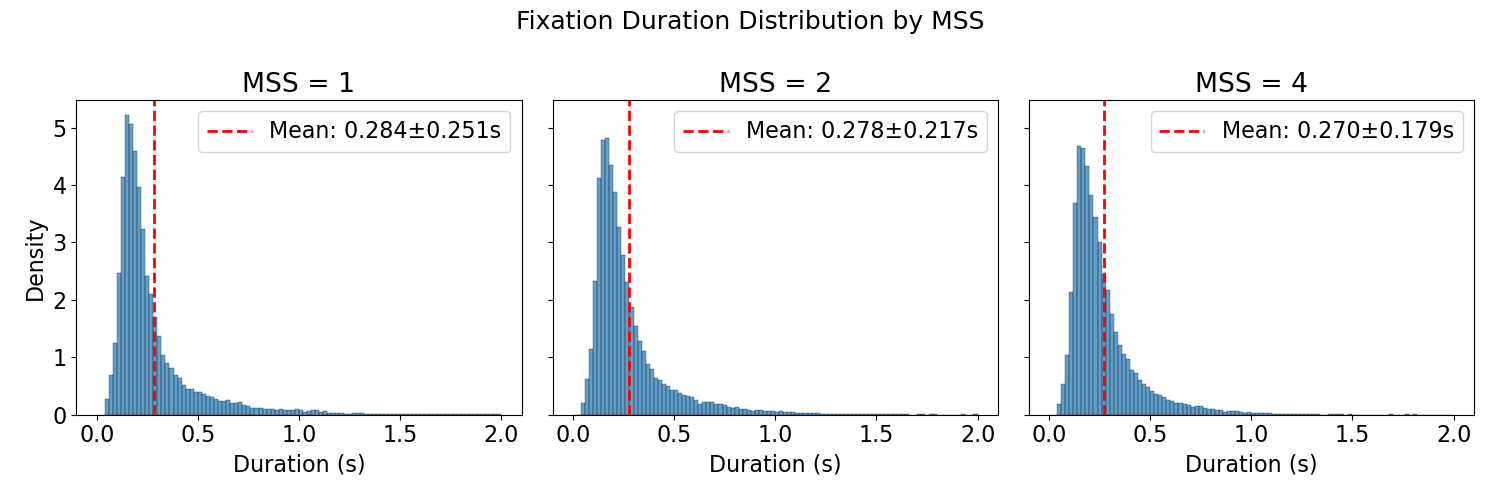

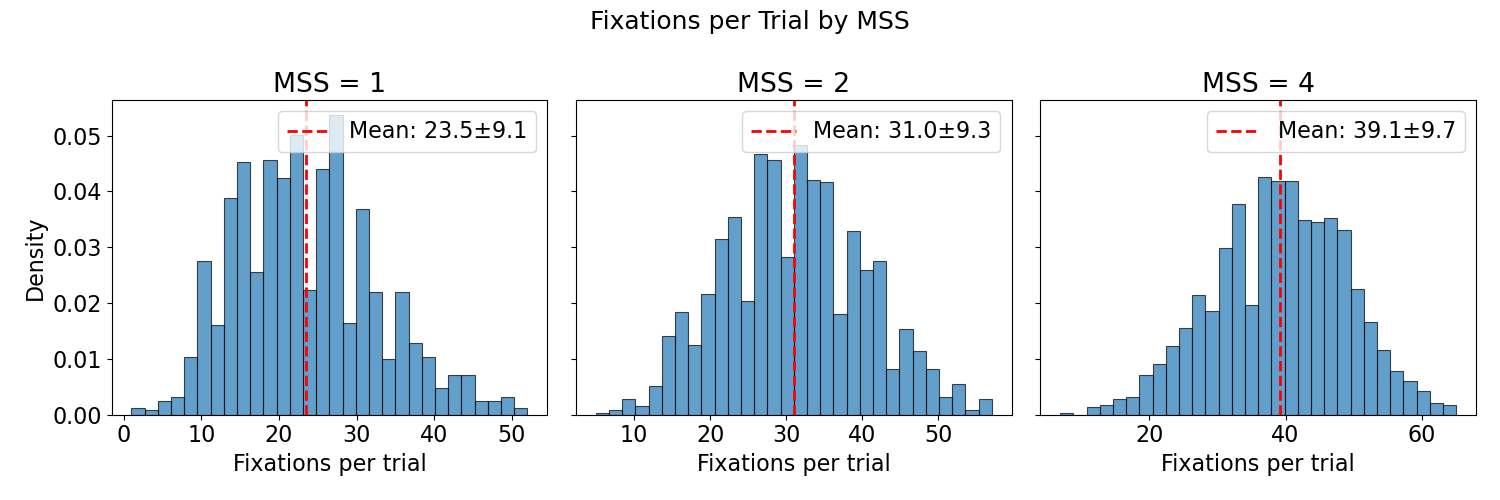


***Supplementary Figure S1:*** *Distribution of saccade amplitude (top) fixation duration (middle) and number of fixations per trial (down), for MSS1 2 and 4. Saccades amplitude and fixations duration show similar distributions and mean values for all MSS. The number of fixations per trial shows an average of approximately 23 for MSS1, 31 for MSS2 and 39 for MSS4.*

## Supplementary Figure S2: Memorization Time-Frequency Response


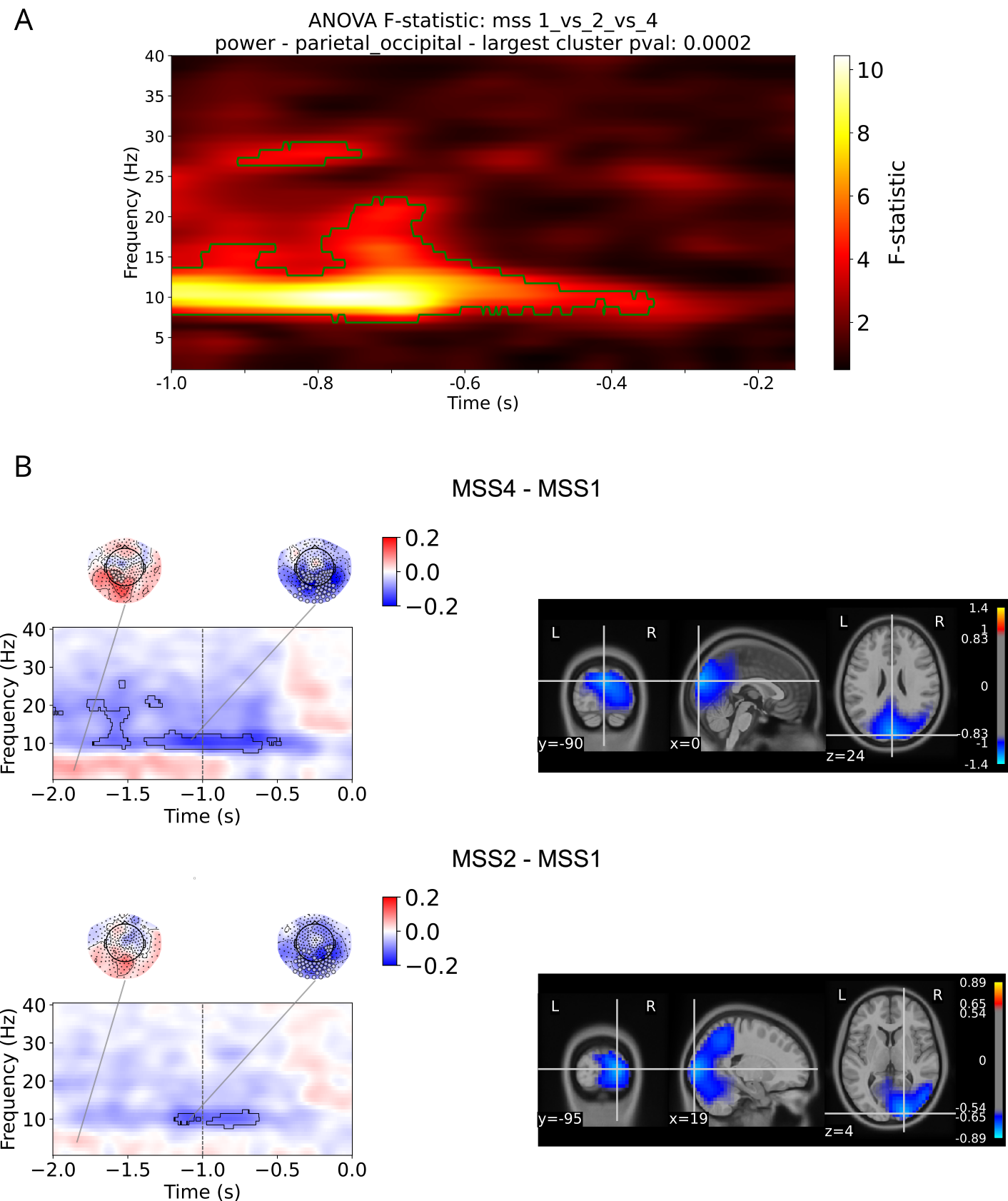


***Supplementary Figure S2:*** *(A) F-statistic of clusters based repeated measures ANOVA across memory load conditions for the retention period, revealing two significant clusters. A large cluster in the alpha band, extending partially to low beta, spanning up to approximately 0.65 s into the retention period (p=0.0002). A second smaller cluster in the beta band, extending until approximately 0.25 s in retention period. (B) Difference between the memory load during memorization.* *Left: temporal frequency response during memory encoding and retention (t = -1 retention period onset). Significant clusters appear mainly in the Alpha band from the end of the encoding period to 0.5 s into the retention period approximately (the largest cluster in both plots has a p-value of 0.00019) Right: Significant power source estimates in the alpha band over the encoding and retention periods (8 to 12 Hz) (p = 0.00019 for MSS2 - MSS1) (p = 0.00019 for MSS4 - MSS1).*

## Supplementary Figure S3: Encoding power and inter-trial coherence (ITC) on correct vs incorrect trials


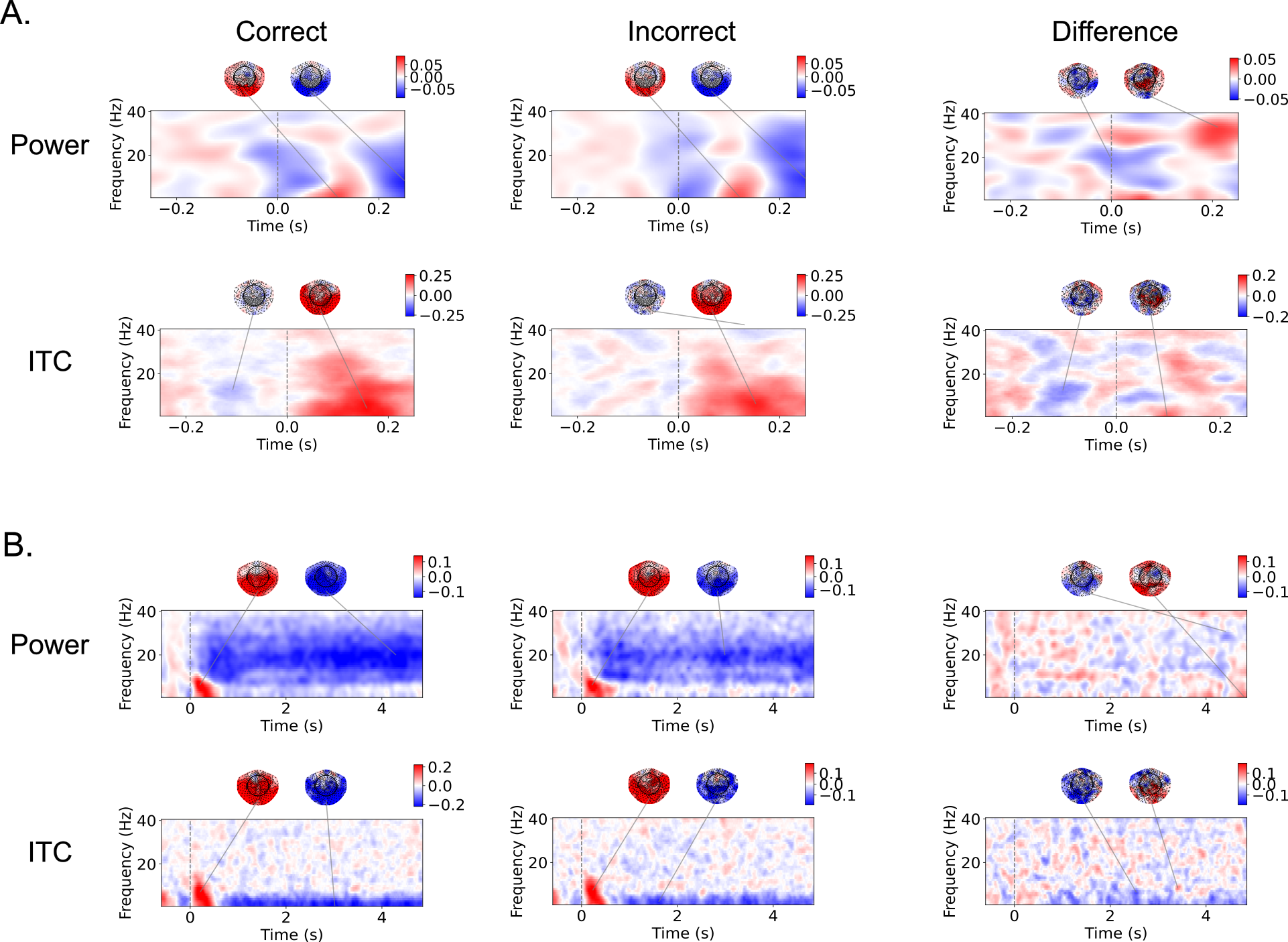


***Supplementary Figure S3:*** *Power and ITC on encoding screen for MSS 4 trials.* ***A.*** *Results aligned to target and distractor saccades for correct and incorrect responses are shown separately alongside the difference between them. No significant differences were found in the difference between conditions.* ***B.*** *Power and ITC aligned to encoding screen onset for correct and incorrect responses are shown separately alongside the difference between them. No significant differences were found between conditions.*

##

## Supplementary Figure S4: Deconvolution on Beta power


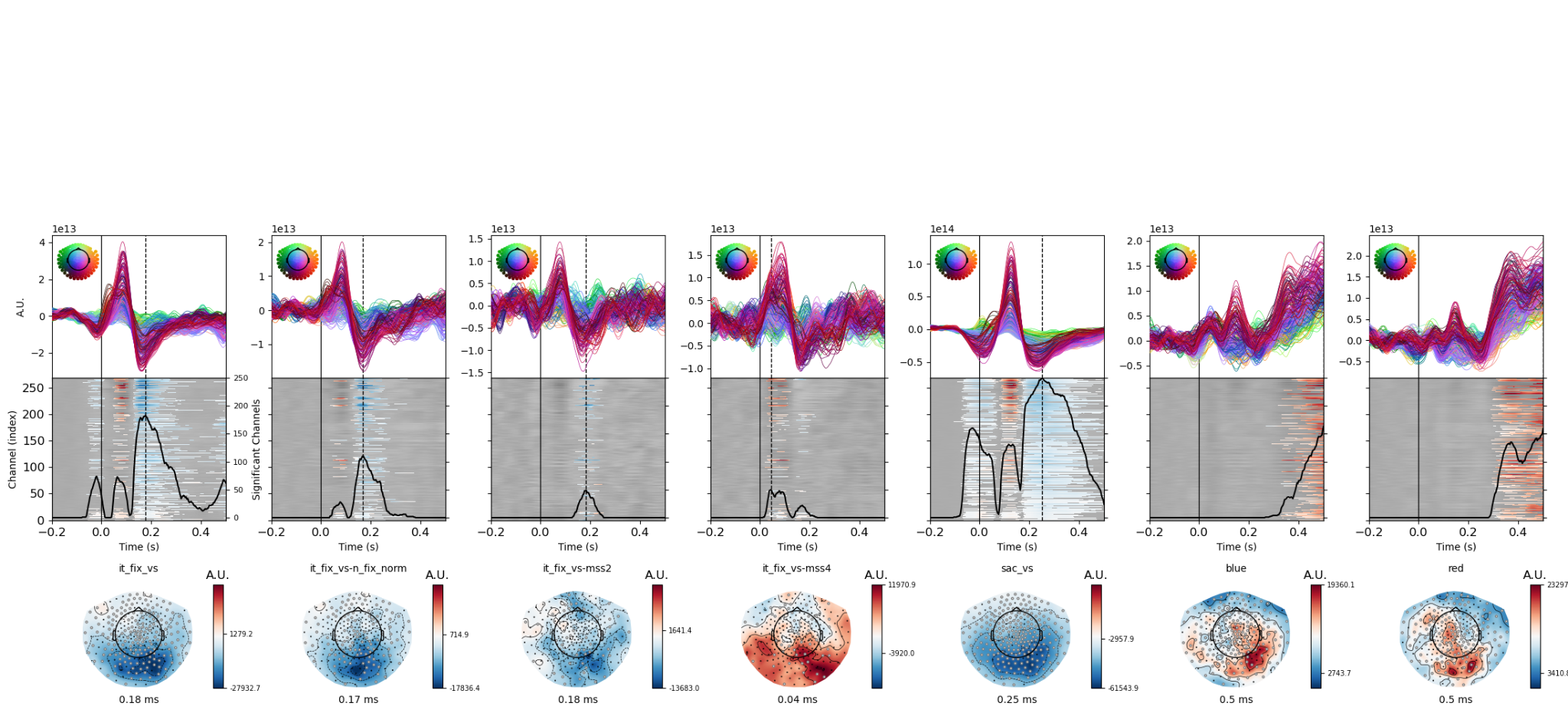


Intercept fix rank MSS2 MSS4 Saccades Left button Right button

***Supplementary Figure S5:*** *Deconvolution analysis on Beta band power. TRF for all features used in the model. From left to right: Top: 1. Fixations onset to distractor items in the visual search screen. 2. Normalized fixation rank, representing the normalized number of fixations in each trial from 0 to 1. 3. Feature indicating 1 for fixations on MSS 2 trials. 4. Feature indicating 1 for fixations on MSS 4 trials. 5. Saccades onset in visual search screen. 6. Left hand button press. 7. Right hand button press. For each feature the top-left panel presents the TRF averaged across participants for all sensors. The middle panel shows the channel's response masking non-significant time-points. The aggregated number of significant sensors over time is shown in black. A black dotted line shows the time with the maximum number of significant sensors. The bottom panel shows the sensor-level topography at the time when the maximum number of significant sensors is found.*

##

## Supplementary Figure S5: Memory load effect on saccades aligned power during visual search

| **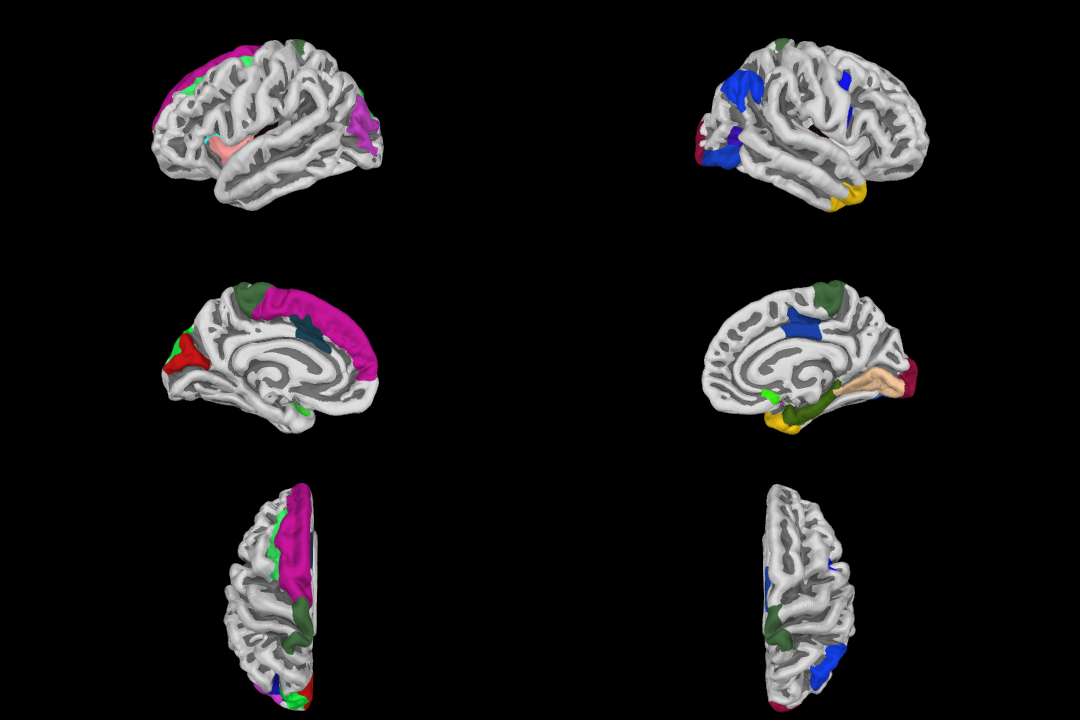** | **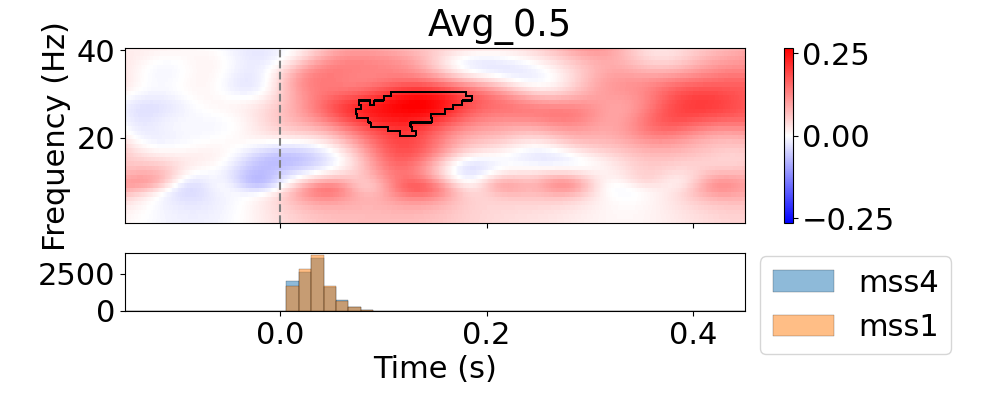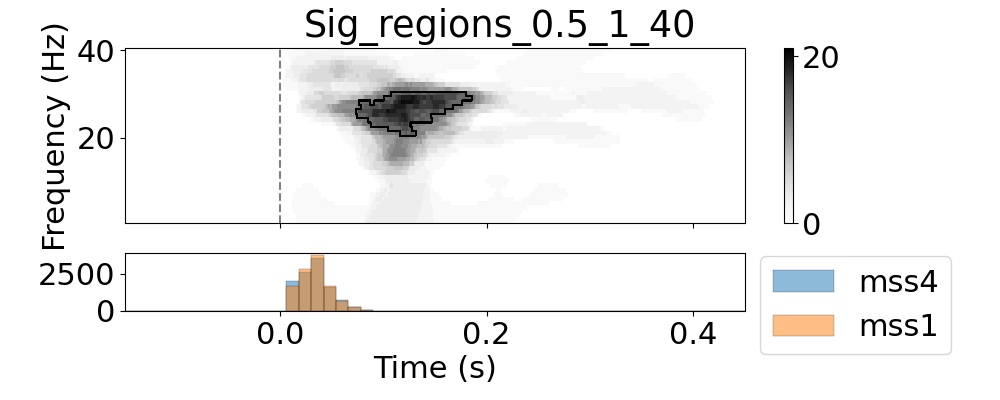** |
| --- | --- |

***Supplementary Figure S4:*** *Working memory load power modulation of saccade response, aligned to saccade onset. Power difference of saccades directed to distractors in visual search of trials under high memory load (MSS=4) and low memory load (MSS=1).*

##

## Supplementary Figure S6: Target response deconvolution analysis

**
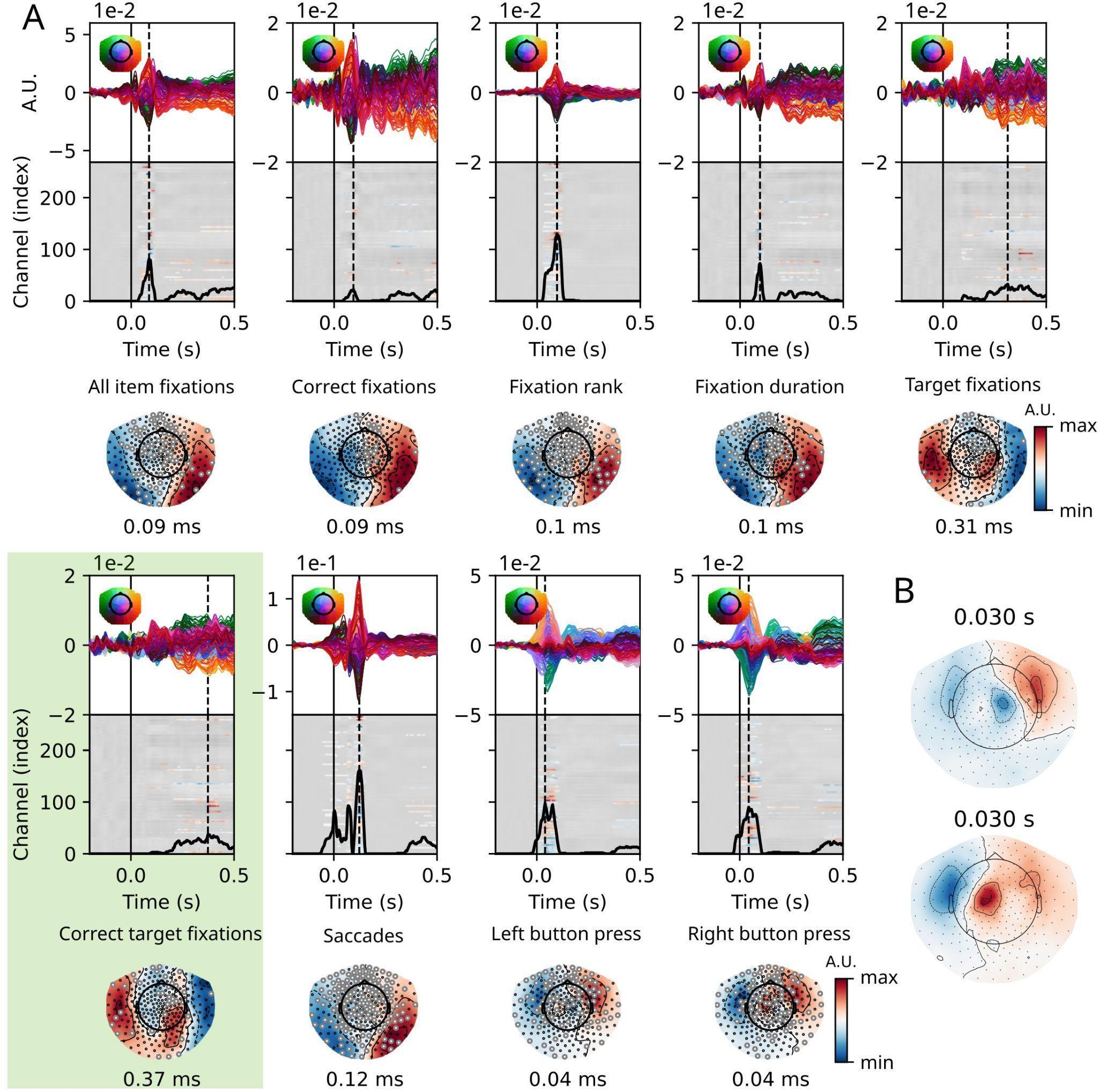
**

***Supplementary Figure S7: Deconvolution analysis. A.*** *TRF for all features used in the model. Top: From left to right: 1. Fixations onset to target and distractor items in the visual search screen. 2. Correct responses for such fixations. 3. Fixations rank for target and distractor fixations. 4. Fixations duration for target and distractor fixations. 5. Fixations onset for target fixations in the visual search screen. Bottom: From left to right: 6. Correct responses for such target fixations. 7. Saccades onset in visual search screen. 8. Left hand button press. 9. Right hand button press. Marked in green is the result from Figure 5B of the manuscript. For each feature the top-left panel presents the TRF averaged across participants for all sensors. The middle panel shows the channel's response masking non-significant time-points. The aggregated number of significant sensors over time is shown in black. A black dotted line shows the time with the maximum number of significant sensors. The bottom panel shows the sensor-level topography at the time when the maximum number of significant sensors is found.* ***B****: Topographic plots of left (top) and right (bottom) button presses at 30ms after the button press, presenting a contralateral response in the central sensors.*

## Supplementary Figure S7: Target-related P3m activations in source space


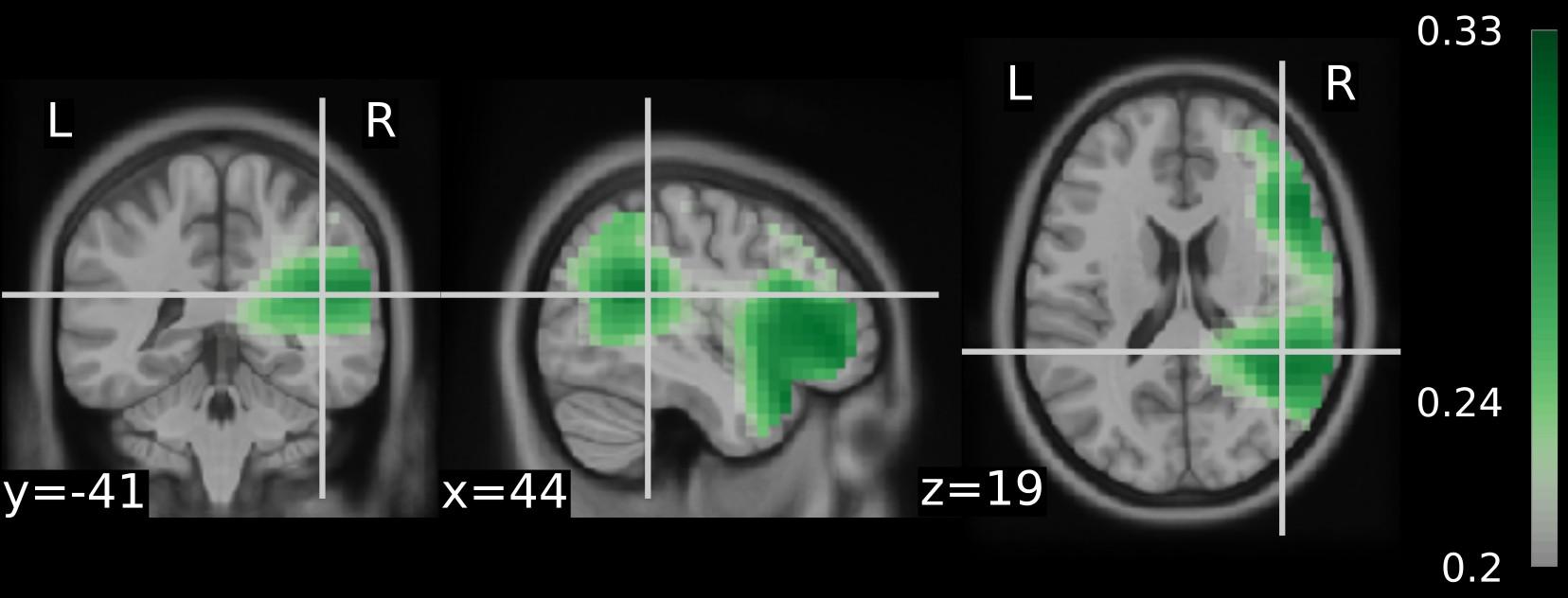


***Supplementary Figure S6****: Activation map showing the cluster of voxels with significantly different responses to target compared to distractor fixations (p-value = 0.00097). The colorbar indicates the duration (in seconds) on which each voxel presented a significant difference between conditions.*

## Supplementary Figure S8: Connectivity analysis


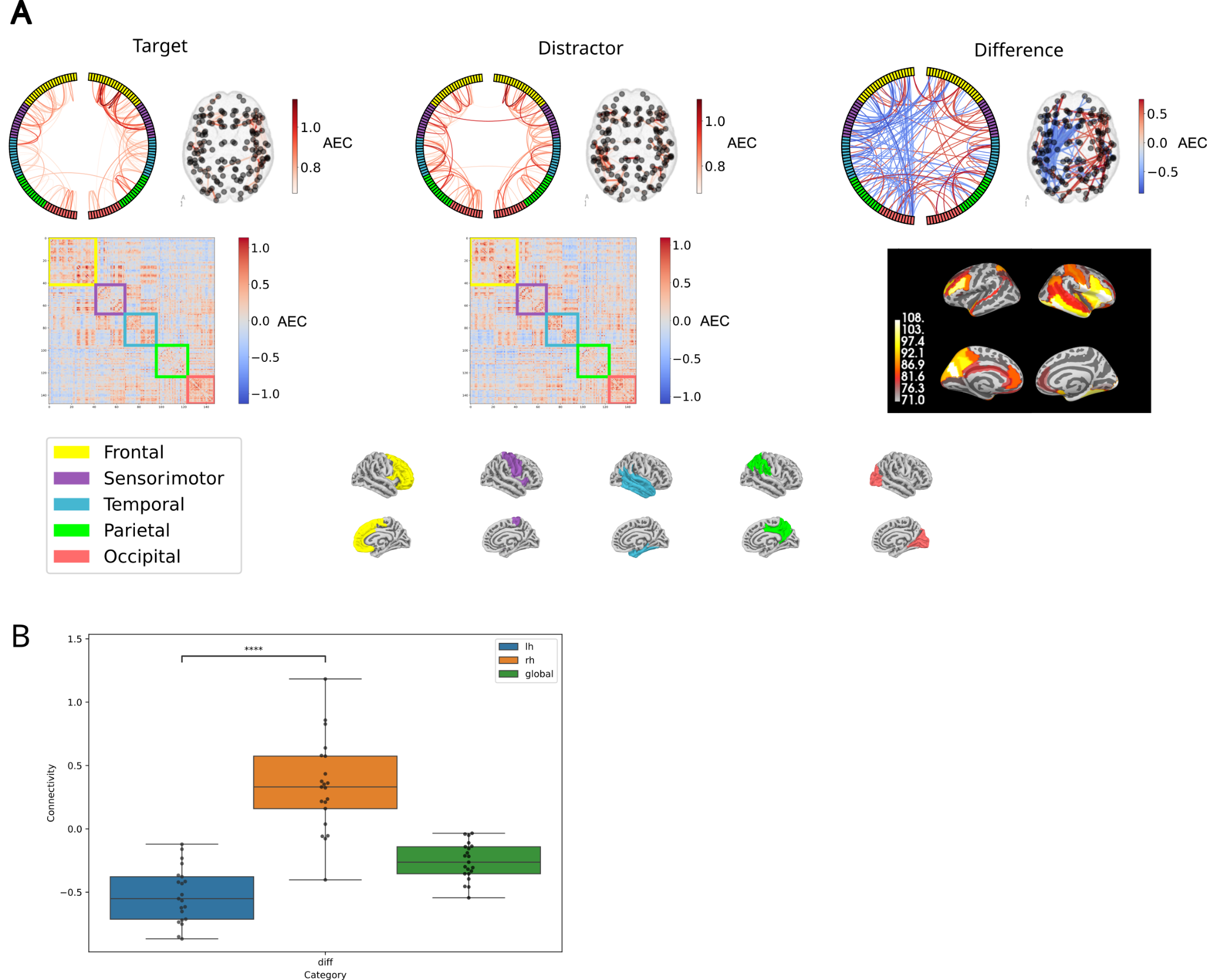


***Supplementary Figure S8: Connectivity analysis*** *(A) Connectome plots, depicted as circular graphs, connectivity matrices, and glass brains, during target and distractor fixations. Values are z-score standardized. The connectome plots show the strongest 150 links from the connectivity matrices. The connectivity degree plots (bottom right) show the sum of the connectivity weights involving each region. (B) Wilcoxon signed-rank test results for connectivity lateralization. Each point in the boxplots corresponds to one subject. For each hemisphere, values represent the mean connectivity strength across the 150 strongest links within the left and right hemisphere, respectively. A paired Wilcoxon signed-rank test between left and right hemispheric connectivity indicated a significant difference (W = 0, p = 0.00000095). One-sample Wilcoxon tests were performed against zero for each hemisphere: Both hemispheres were significantly different from zero (W = 0, p = 0.00000095 for left hemisphere), (W = 23, p = 0.0006 for right hemisphere).*
